# Inhibition of CILP2 Improves Glucose Metabolism and Mitochondrial Dysfunction in Sarcopenia via the Wnt Signalling Pathway

**DOI:** 10.1002/jcsm.13597

**Published:** 2024-10-10

**Authors:** Zhibo Deng, Chao Song, Long Chen, Rongsheng Zhang, Linhai Yang, Peng Zhang, Yu Xiu, Yibin Su, Fenqi Luo, Jun Luo, Hanhao Dai, Jie Xu

**Affiliations:** ^1^ Shengli Clinical Medical College Fujian Medical University Fuzhou People's Republic of China; ^2^ Department of Orthopedics, Fujian Provincial Hospital Fujian Medical University Fuzhou People's Republic of China; ^3^ College of Traditional Chinese Medicine Fujian University of Traditional Chinese Medicine Fuzhou People's Republic of China

**Keywords:** CILP2, glucose metabolism, insulin resistance, sarcopenia, Wnt/β‐catenin pathway

## Abstract

**Background:**

Skeletal muscle is the primary organ involved in insulin‐mediated glucose metabolism. Elevated levels of CILP2 are a significant indicator of impaired glucose tolerance and are predominantly expressed in skeletal muscle. It remains unclear whether CILP2 contributes to age‐related muscle atrophy through regulating the glucose homeostasis and insulin sensitivity.

**Methods:**

Initially, the expression levels of CILP2 were assessed in elderly mice and patients with sarcopenia. Lentiviral vectors were used to induce either silencing or overexpression of CILP2 in C2C12 myoblast cells. The effects of CILP2 on proliferation, myogenic differentiation, insulin sensitivity and glucose uptake were evaluated using immunofluorescence, western blotting, real‐time quantitative polymerase chain reaction, RNA sequencing, glucose uptake experiments, dual‐luciferase reporter assays and co‐immunoprecipitation (CO‐IP). An adeno‐associated virus‐9 containing a muscle‐specific promoter was injected into SAMP8 senile mice to observe the efficacy of CILP2 knockout.

**Results:**

We found that there was more CLIP2 expressed in the skeletal muscle of ageing mice (+1.1‐fold, *p* < 0.01) and in patients with sarcopenia (+2.5‐fold, *p* < 0.01) compared to the control group. Following the overexpression of CILP2, Ki67 (−65%, *p* < 0.01), PCNA (−32%, *p* < 0.05), MyoD1 (−89%, *p* < 0.001), MyoG (−31%, *p* < 0.05) and MyHC (−85%, *p* < 0.001), which indicate proliferation and differentiation potential, were significantly reduced. In contrast, MuRF‐1 (+59%, *p* < 0.05), atrogin‐1 (+43%, *p* < 0.05) and myostatin (+31%, *p* < 0.05), the markers of muscular atrophy, were significantly increased. Overexpression of CILP2 decreased insulin sensitivity, glucose uptake (−18%, *p* < 0.001), GLUT4 translocation to the membrane and the maximum respiratory capacity of mitochondria. Canonical Wnt signalling was identified through RNA sequencing as a potential pathway for CILP2 regulation in C2C12, and Wnt3a was confirmed as an interacting protein of CILP2 in the CO‐IP assay. The addition of recombinant Wnt3a protein reversed the inhibitory effects on myogenesis and glucose metabolism caused by CILP2 overexpression. Conversely, CILP2 knockdown promoted myogenesis and glucose metabolism. CILP2 knockdown improved muscle atrophy in mice, characterized by significant increases in time to exhaustion (+42%, *p* < 0.001), grip strength (+19%, *p* < 0.01), muscle mass (+15%, *p* < 0.001) and mean muscle cross‐sectional area (+37%, *p* < 0.01). CILP2 knockdown enhanced glycogen synthesis (+83%, *p* < 0.001) and the regeneration of oxidative and glycolytic muscle fibres in SAMP8 ageing mice via the Wnt/β‐catenin signalling pathway.

**Conclusions:**

Our results indicate that CILP2 interacts with Wnt3a to suppress the Wnt/β‐catenin signalling pathway and its downstream cascade, leading to impaired insulin sensitivity and glucose metabolism in skeletal muscle. Targeting CILP2 inhibition could offer potential therapeutic benefits for sarcopenia.

## 1 Introduction

Sarcopenia, a prevalent condition among the elderly, is a growing but often underappreciated health issue characterized by a progressive decline in skeletal muscle mass, strength and function [1, 2]. It is linked to a higher incidence of various adverse health outcomes, including falls, fractures, decreased functionality, frailty, disability and mortality [3, 4]. The prevalence of sarcopenia is estimated to be around 14% in individuals aged 65–70 years and can reach as high as 53% in those over 80 years. As the global population ages, addressing sarcopenia and mitigating mobility disorders in the elderly has become urgent [5].

In addition to being closely linked with ageing, sarcopenia is also believed to be both a cause and a consequence of diabetes [6, 7]. As the primary organ for glucose absorption, the loss of muscle mass and impaired function associated with sarcopenia hinders skeletal muscle from processing glucose, thereby increasing the risk of diabetes in individuals with sarcopenia [8]. As the primary organ for glucose absorption, the loss of muscle mass and impaired function associated with sarcopenia hinders skeletal muscle from processing glucose, thereby increasing the risk of diabetes in individuals with sarcopenia [9]. Mitochondrial dysfunction in skeletal muscle cells is considered another significant factor in sarcopenia [10]. Beyond ATP energy production, mitochondria also regulate intracellular calcium homeostasis and apoptosis signalling in skeletal muscle fibres [11, 12]. Additionally, mitochondria are involved in metabolic reprogramming in sarcopenia, leading to impaired uptake and utilization of glucose, protein and fat [13]. However, current research on sarcopenia has primarily concentrated on increasing muscle mass rather than metabolism or function. Furthermore, unspecified antidiabetic drugs may have either harmful or beneficial effects on skeletal muscle. Therefore, a deeper understanding of glucose metabolism in skeletal muscle is essential [14].

In recent years, Massart et al. [15] has reported that miR‐19b‐3p produced by aerobic exercise can enhance insulin signalling, glucose uptake and maximal oxygen consumption in skeletal muscle cells, thereby modifying gene expression and protein abundance in skeletal muscle [16]. Aside from exercise, little is known about the mechanisms involved in sarcopenia and glucose metabolism [17]. The cartilage intermediate layer protein (CILP) family, which consists of CILP1 and CILP2, is primarily distributed in cartilage cells but can also be expressed in various other tissues. The expression levels in the central nervous system, heart and skeletal muscle are relatively high [18, 19]. Li et al. [20] found that serum CILP2 concentrations in the ultra‐obese and obese groups were significantly higher than those in the normal weight group. Bioinformatic analysis of CILP2 protein interactions has indicated that CILP2‐related genes are involved mainly in energy metabolism, skeletal muscle phylogenesis and insulin resistance (IR). Wu et al. [21] discovered that CILP2 is elevated in obese and diabetic patients, primarily in muscle, and that overexpression of CILP2 inhibits glucose metabolism in the liver by directly acting on PEPCK (a key gluconeogenic enzyme), while also impacting insulin sensitivity in skeletal muscle. Owing to the complexity and interrelationships of these molecular pathways, it remains unclear whether CILP2 plays a role in sarcopenia through the regulation of glucose homeostasis and IR.

## 2 Methods

### 2.1 Animals Studies

C57BL/6 and senescence‐accelerated mouse P8 (SAMP8) male mice were purchased from the Fujian Provincial Hospital Laboratory Animal Center, and SAMP8 mice were not tested until they reached 8 months of age [22]. SAMP8 mice have been extensively utilized as an age‐associated sarcopenia model in studies focused on muscle atrophy [23, 24, 25]. They were housed in a controlled environment with suitable temperature and humidity levels, under a 12‐h light/12‐h dark cycle, and given ad libitum access to food and tap water. Adeno‐associated virus 9 (AAV9) vectors encoding sh‐CILP2 (AAV9‐eGFP‐MYOGp‐sh‐CILP2) and AAV9 vectors encoding scramble (AAV9‐eGFP‐MYOGp‐sh‐Scramble; sh‐Scramble) were constructed by Genechem Company (Shanghai, China), labelled with eGFP and containing muscle‐specific promoters. Eighteen 8‐month‐old male SAMP8 mice were randomly divided into three groups as follows: (1) In the Control group, 20‐μL PBS was injected multifocally into the bilateral gastrocnemius (Control, *n* = 6); (2) in the AAV9‐sh‐Scramble group, sh‐Scramble at 2 × 10^11^ vector genomes (vg)/GA was diluted to 20 μL with PBS and then injected into the bilateral gastrocnemius at multiple sites (sh‐Scramble, *n* = 6); (3) in the AAV9‐sh‐CILP2 group, sh‐CILP2 was injected following the same protocol as the sh‐Scramble group (sh‐CILP2, *n* = 6). Behavioural testing commenced 2 months after AAV9 injection. As in the previous study [26], the mice were subjected to a treadmill (Treadmill TSE Systems, Germany) for exercise tolerance test. The hindlimb grip strength was measured using a Grip Strength Meter (XinRuan, Shanghai, China). After the experiment, the mice were euthanized in a CO_2_ chamber. We conducted histological and immunofluorescence (IF) analyses as previously described [27], with modifications outlined in the Methods section of the Supporting Information. The Ethics of Animal Experiments Committee at Fujian Provincial Hospital approved all animal procedures (IACUC‐FPH‐SL‐20240228[0121]), and all animals were cared for in accordance with the Guide for the Care and Use of Laboratory Animals.

### 2.2 Cell Culture

Mouse myoblasts C2C12 cell lines were provided by Cell bank, Chinese Academy of Sciences (China). Cell culture and differentiation were performed as previously described [28]. The cell culture utilized Dulbecco's Modified Eagle Medium (DMEM)–high glucose (ThermoFisher), supplemented with 10% foetal bovine serum and 1% penicillin/streptomycin. Differentiation medium was prepared by adding 2% horse serum (16050122, ThermoFisher, USA) and 1% penicillin/streptomycin to the DMEM. Cultures were maintained in an incubator at 37°C with 5% CO_2_. Once cell confluence reached approximately 80%, the differentiation medium replaced the growth medium to promote myogenic differentiation. The medium was replenished every 2 days. The C2C12 myoblast differentiation model was established through continuous induction and differentiation over a period of 6 days, subsequently employed for biochemical analyses. The insulin stimulation test was performed by adding 100‐mM insulin 30 min prior to protein extraction.

### 2.3 Additional Methods

Additional details regarding the methods and materials are provided in the Supporting Information.

### 2.4 Statistical Analysis

Data were analysed using GraphPad Prism 9.0 software (9.0, Graph Software, USA). All data are expressed as mean ± standard deviation (SD). The normality of distribution was assessed using the Shapiro–Wilk test. Comparisons between two groups were performed with Student's *t* tests or a non‐parametric Mann–Whitney test. For comparisons involving more than two groups, statistical significance was calculated using one‐way analysis of variance (ANOVA) with Tukey's post hoc test. A parametric two‐way ANOVA was employed for multiple comparisons involving distinct groups across various outcomes. *p* < 0.05 indicates significance.

## 3 Results

### 3.1 Glycogen Levels in Skeletal Muscle Significantly Decrease With Age

Since ageing encompasses multiple biological processes, we first assessed the capacity for glycogen synthesis in vivo in both sarcopenia patients and elderly mice. We enrolled four patients with sarcopenia and four without. From the demographic information in Table S4, it is evident that grip strength (10.40 ± 1.13 vs. 25.88 ± 3.19 kg, *p* < 0.001) and Skeletal Muscle Mass Index (4.38 ± 0.38 vs. 6.98 ± 1.12 kg/m^2^, *p* < 0.01) in the sarcopenia group were significantly lower than in the non‐sarcopenia group. Laboratory results also indicated that sarcopenia patients exhibited higher levels of inflammation. HE staining (Figure S1A) and Masson staining (Figure S1B) revealed a significant decrease in the CSA of the quadriceps muscle in sarcopenia patients, along with an increase in fibre area. PAS staining (Figure S1C) and glycogen content (Figure S1D) were significantly reduced in sarcopenia patients compared to those without. IF staining demonstrated a decrease in the proportion of type II muscle fibres and an increase in type I muscle fibres in the skeletal muscle of sarcopenia patients (Figure S1E). In C57BL/6J mice, HE staining (Figure S1F) and Masson staining (Figure S1G) showed a significant reduction in CSA in GA muscle and an increase in fibre area in 24‐month‐old mice. Compared to 3‐month‐old young mice, PAS staining and glycogen content in 24‐month‐old mice were significantly lower (Figure S1H,I). IF staining also indicated a decrease in the proportion of type II muscle fibres and an increase in type I muscle fibres in the 24‐month‐old mice (Figure S1J). These findings suggest that reduced glycogen synthesis may be linked to sarcopenia.

### 3.2 CILP2 Is Upregulated in Muscle During Sarcopenia

**FIGURE 1:**
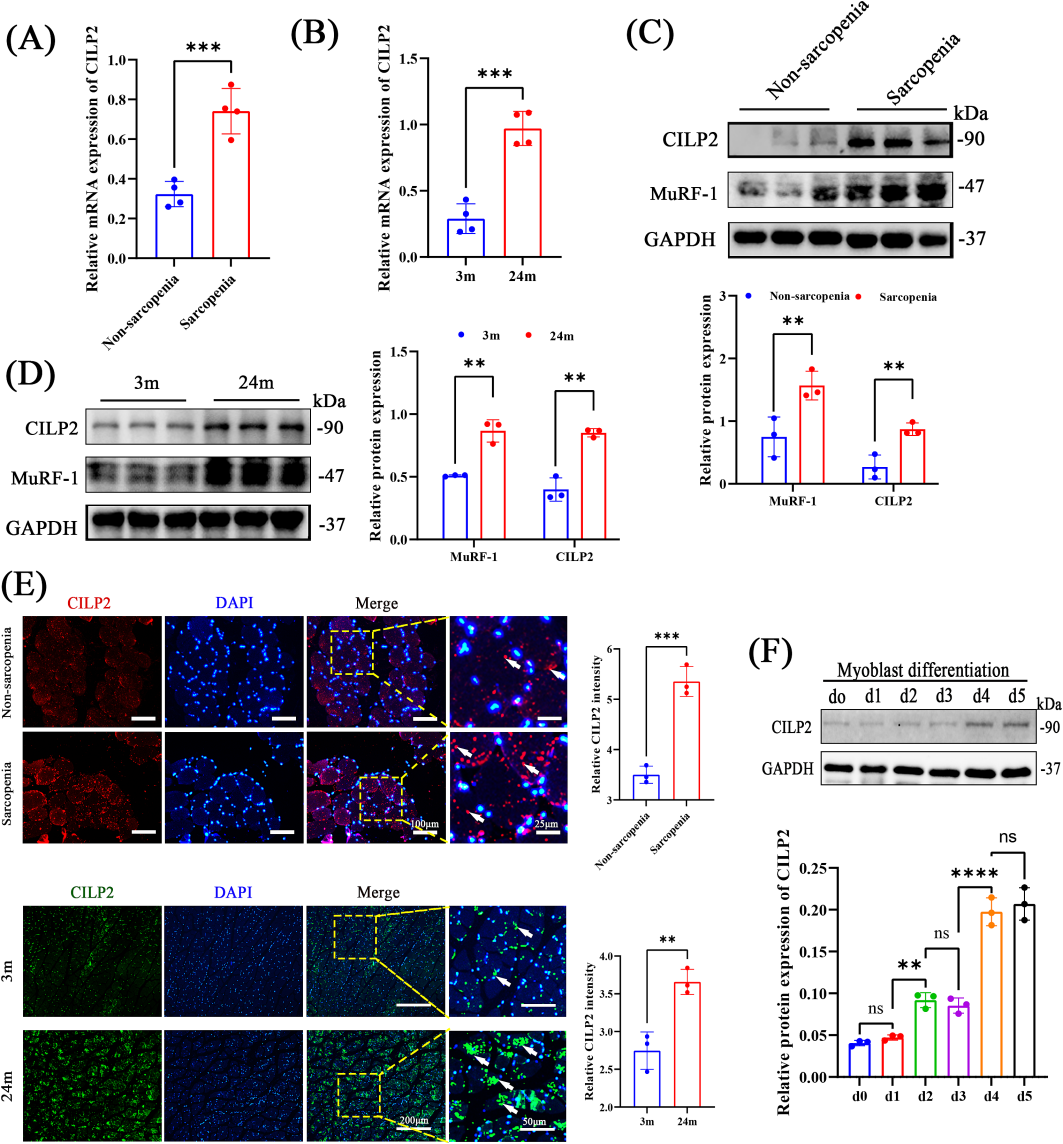
CILP2 is upregulated in skeletal muscle of patients with sarcopenia and elderly mice. (A) The mRNA levels of CILP2 in quadriceps muscle of non‐sarcopenia and sarcopenia, *n* = 4. (B) The mRNA levels of CILP2 in GAs muscle of 3‐ and 24‐month‐old mice, *n* = 4. (C) Western blotting and quantitative analysis of the levels of CILP2 and MuRF‐1 in non‐sarcopenia and sarcopenia, *n* = 3. (D) Western blotting and quantitative analysis of the levels of CILP2 and MuRF‐1 in 3‐ and 24‐month‐old mice, *n* = 3. (E) Representative IF staining and the fluorescence density of CILP2 in non‐sarcopenia and sarcopenia and in 3‐ and 24‐month‐old mice, scale bars = 100 and 200 μm. (F) Western blotting and quantitative analysis of the levels of CILP2 during myoblast differentiation, *n* = 3. (E) The positive areas are indicated by white arrows. (E) *n* = 3, one fields per sample were selected. For all statistical plots, values are shown as mean ± SD. Ns, no significance, **p < 0.01, ***p < 0.001, ****p < 0.0001. Statistical significance was determined by Student's *t* test (for A, B, D and E) or one‐way ANOVA (for F). GA, gastrocnemius.

We evaluated CILP2 expression patterns during ageing and the progression of sarcopenia to determine whether CILP2 is involved in skeletal muscle regeneration and glucose metabolism. We analysed the mRNA and protein levels of CILP2 in non‐sarcopenic and sarcopenic conditions using qRT‐PCR (Figure 1A), western blotting (WB) (Figure 1C) and IF (Figure 1E), respectively. CILP2 expression was significantly elevated in the skeletal muscle of patients with sarcopenia compared to those without. Similarly, we demonstrated this finding in C57BL/6J mice in the senescence model (24 months old). qRT‐PCR (Figure 1B), WB (Figure 1D) and IF (Figure 1E) revealed that the expression level of CILP2 in ageing mice was also significantly increased compared to that of young 3‐month‐old mice. Additionally, we found that the protein level of CILP2 was strongly increased during myoblast differentiation (Figure 1F), indicating that CILP2 is associated with myogenesis. These results strongly suggest that CILP2 may be involved in the progression of age‐related muscle physiology.

### 3.3 CILP2 Overexpression Impairs the Proliferation and Differentiation Potential of C2C12 Myoblasts

**FIGURE 2:**
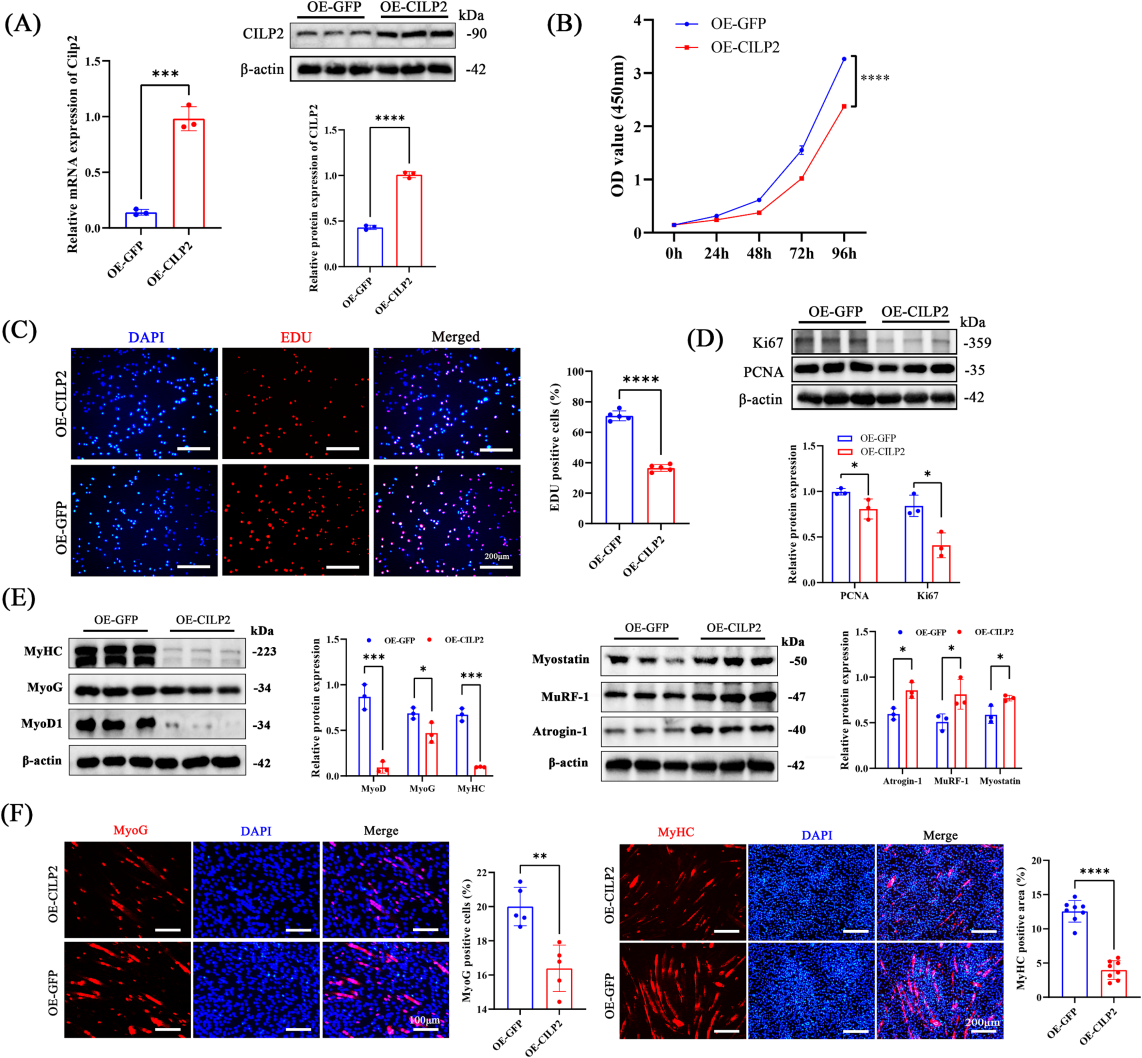
Overexpression of CILP2 inhibits proliferation and differentiation potential of C2C12 myoblasts. (A) At 48 h post‐transfection, the mRNA and protein levels of CILP2 in OE‐GFP and OE‐CILP2, *n* = 3. (B) CCK8 assay for cell proliferation of the OE‐GFP and OE‐CILP2, *n* = 3. (C) Representative EdU staining of the OE‐GFP and OE‐CILP2 and positive cells counting, scale bars = 200 μm. (D) Western blotting and quantitative analysis of the levels of PCNA and Ki67 in OE‐GEP and OE‐CILP2, *n* = 3. (E) Western blotting and quantitative analysis of the levels of MyoD1, MyoG, MyHC, Atrogin‐1, MuRF‐1 and Myostatin in OE‐GEP and OE‐CILP2, *n* = 3. (F) Representative IF staining of MyoG and MyHC in OE‐GFP and OE‐CILP2, scale bars = 100 and 200 μm. (C) *n* = 5, one fields per sample were selected. (F) *n* = 4, two fields per sample were selected. For all statistical plots, values are shown as mean ± SD. *p < 0.05, **p < 0.01, ***p < 0.001, ****p < 0.0001. Statistical significance was determined by Student's *t* test (for A, B, C, D, E and F).

To further elucidate the role of CILP2 in satellite cell proliferation and myogenic differentiation, CILP2‐eGFP lentiviral vectors were constructed for CILP2 overexpression (OE‐CILP2) or control (OE‐GFP) in C2C12 cell lines. qRT‐PCR and WB (Figure 2A) demonstrated that CILP2 was successfully overexpressed. In the CCK8 and EDU proliferation assays (Figure 2B,C), OE‐CILP2 inhibited cell proliferation. Similarly, the protein levels of the proliferation‐related proteins Ki67 and PCNA in OE‐CILP2 cells were significantly reduced (Figure 2D). Following differentiation induction, OE‐CILP2 significantly decreased MyoG, MyoD1 and MyHC (Figure 2E). During muscle atrophy, atrogene E3 ubiquitin ligases are upregulated and bind ubiquitin to various muscle proteins for proteasome degradation [29]. We found that OE‐CILP2 significantly increased E3 ubiquitin ligases (Atrogin‐1 and MuRF‐1) and Myostatin (Figure 2E). Additionally, IF analysis of MyoG and MyHC (Figure 2F) indicated that myogenic differentiation was impaired and that the cell fusion index significantly decreased after CILP2 overexpression.

### 3.4 CILP2 Deficiency Promotes the Proliferation and Myogenic Differentiation of C2C12 Myoblasts

**FIGURE 3:**
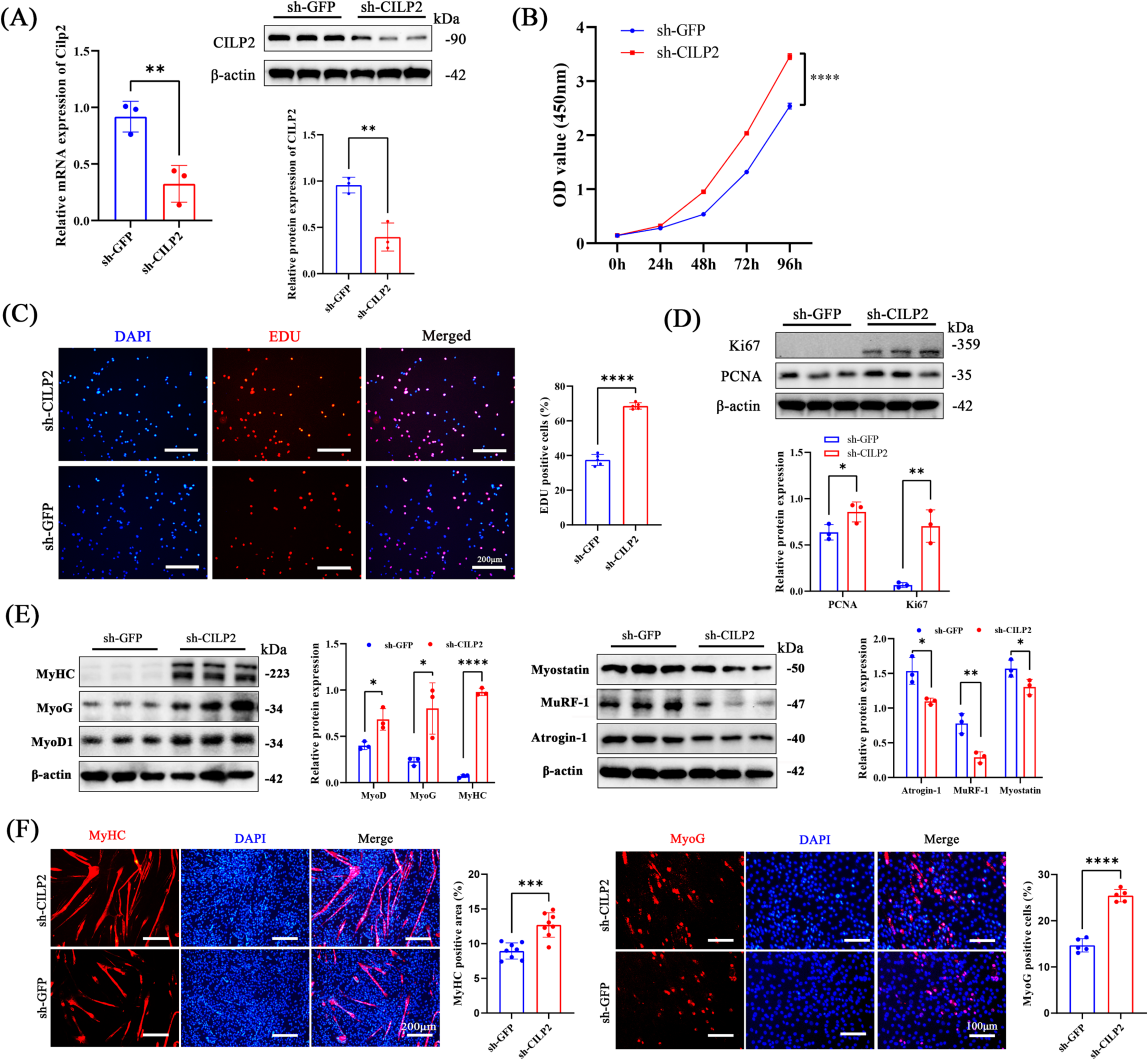
CILP2 knockout promotes proliferation and differentiation potential of C2C12 myoblasts. (A) At 48 h post‐transfection, the mRNA and protein levels of CILP2 in sh‐GFP and sh‐CILP2, *n* = 3. (B) CCK8 assay for cell proliferation of the sh‐GFP and sh‐CILP2, *n* = 3. (C) Representative EdU staining of the sh‐GFP and sh‐CILP2 and positive cells counting, scale bars = 200 μm. (D) Western blotting and quantitative analysis of the levels of PCNA and Ki67 in sh‐GFP and sh‐CILP2, *n* = 3. (E) Western blotting and quantitative analysis of the levels of MyoD1, MyoG, MyHC, Atrogin‐1, MuRF‐1 and Myostatin in sh‐GFP and sh‐CILP2, *n* = 3. (F) Representative IF staining of MyoG and MyHC in sh‐GFP and sh‐CILP2, scale bars = 100 and 200 μm. (C) *n* = 5, one fields per sample were selected. (F) *n* = 4, two fields per sample were selected. For all statistical plots, values are shown as mean ± SD. *p < 0.05, **p < 0.01, ***p < 0.001, ****p < 0.0001. Statistical significance was determined by Student's *t* test (for A, B, C, D, E and F).

To further verify the precise role of CILP2 in skeletal muscle, CILP2‐eGFP lentiviral vectors were constructed for CILP2 silencing (sh‐CILP2) or control (sh‐GFP) in C2C12 cell lines. Both qRT‐PCR and WB (Figure 3A) demonstrated that CILP2 silencing significantly reduced CILP2 expression. CCK8 and EDU proliferation assays showed that sh‐CILP2 enhanced the proliferation of C2C12 cells (Figure 3B,C). Additionally, the protein levels of Ki67 and PCNA in sh‐CILP2 cells were significantly increased (Figure 3D). Following differentiation induction, sh‐CILP2 elevated MyoG, MyoD1 and MyHC levels (Figure 3E), while significantly decreasing Atrogin‐1, MuRF‐1 and Myostatin (Figure 3E). Furthermore, IF analysis of MyoG and MyHC (Figure 3F) revealed a significant increase in myotube formation and the cell fusion index in sh‐CILP2 group.

### 3.5 CILP2 Deficiency Promotes Mitochondrial Biogenesis in Myotubes

**FIGURE 4:**
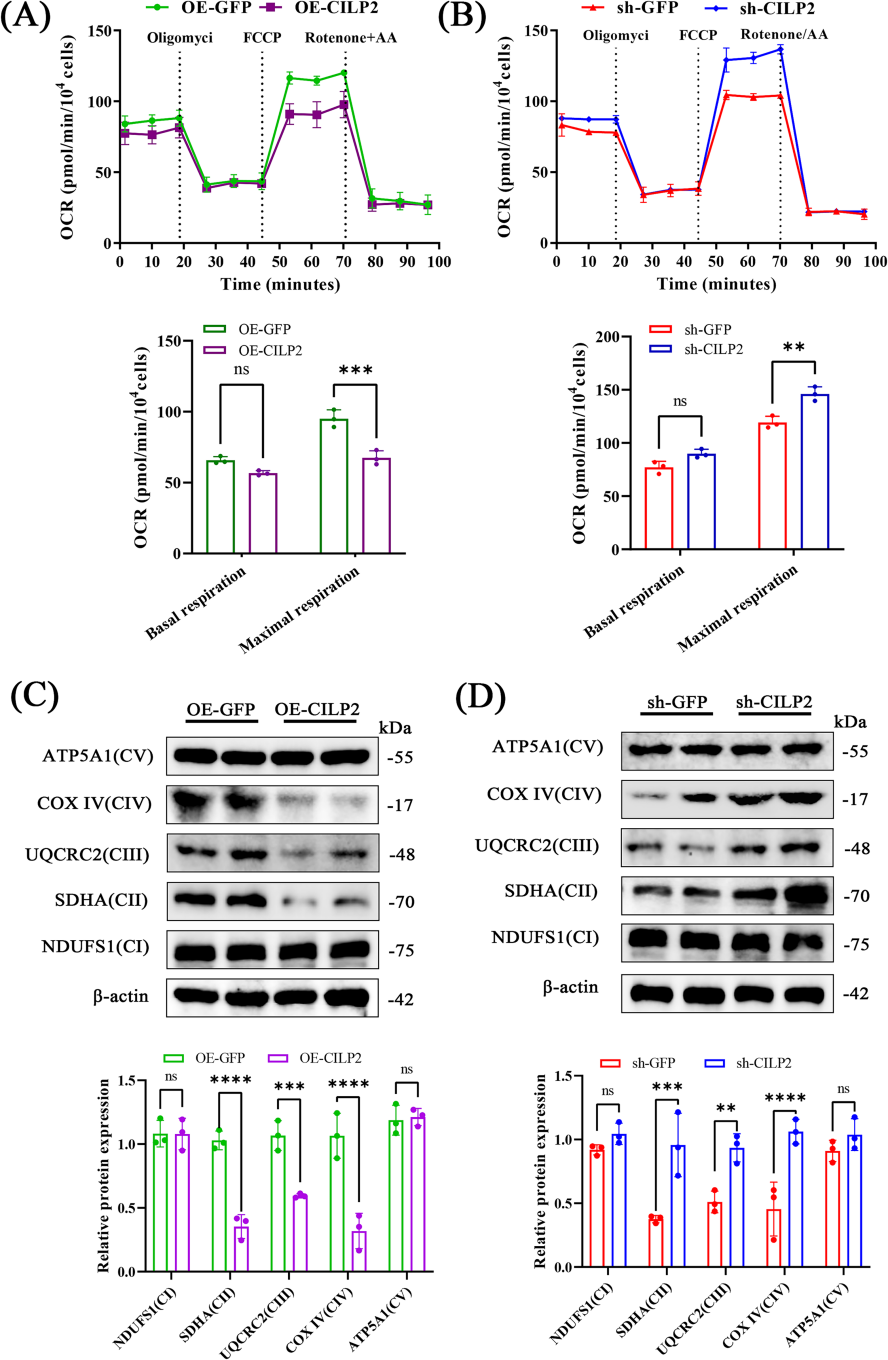
Upregulation of CILP2 inhibits mitochondrial biogenesis in myotube. (A) The graphs of mitochondrial respiration by OCR quantification presented basal respiration, and maximal respiration in OE‐GFP and OE‐CILP2 myotube, *n* = 3. (B) The graphs of mitochondrial respiration by OCR quantification presented basal respiration and maximal respiration in sh‐GFP and sh‐CILP2 myotube, *n* = 3. (C) Western blotting and quantitative analysis of OXPHOS complexes (NDUFS1, SDHA, UQCRC2, COX IV and ATP5A1) in OE‐GFP and OE‐CILP2 myotube, *n* = 3. (D) Western blotting and quantitative analysis of OXPHOS complexes (NDUFS1, SDHA, UQCRC2, COX IV and ATP5A1) in sh‐GFP and sh‐CILP2 myotube, *n* = 3. For all statistical plots, values are shown as mean ± SD. Ns, no significance, **p < 0.01, ***p < 0.001, ****p < 0.0001. Statistical significance was determined by Student's *t* test (for A, B, C, D and E).

Considering that the differentiation status of skeletal muscle affects metabolism [30], we further verified the role of CILP2 in muscle metabolism. Since mitochondria are the primary sites of metabolism, we used the OCR to assess mitochondrial respiratory capacity in C2C12 cells. OE‐CILP2 was found to inhibit myotube maximal respiration (FCCP‐stimulated) (Figure 4A), while CILP2 ablation increased myotube maximal respiration (Figure 4B). Additionally, we detected changes in the abundance of mitochondrial complex subunits. The overexpression of CILP2 was accompanied by a decrease in the abundance of SDHA (complex II subunit), UQCRC2 (complex III subunit) and COX IV (complex IV subunit), indicating that oxidative phosphorylation was inhibited (Figure 4C). In contrast, we found that silencing CILP2 increased the abundance of complex II and complex IV (Figure 4D). NDUFS1 of complex I and ATP5A1 of complex V did not significantly change. Thus, the knockout of CILP2 increases the abundance of mitochondrial protein complexes and promotes mitochondrial biogenesis in myotubes.

### 3.6 Increased CILP2 Impairs Glucose Metabolism and Insulin Sensitivity in Myotubes

**FIGURE 5:**
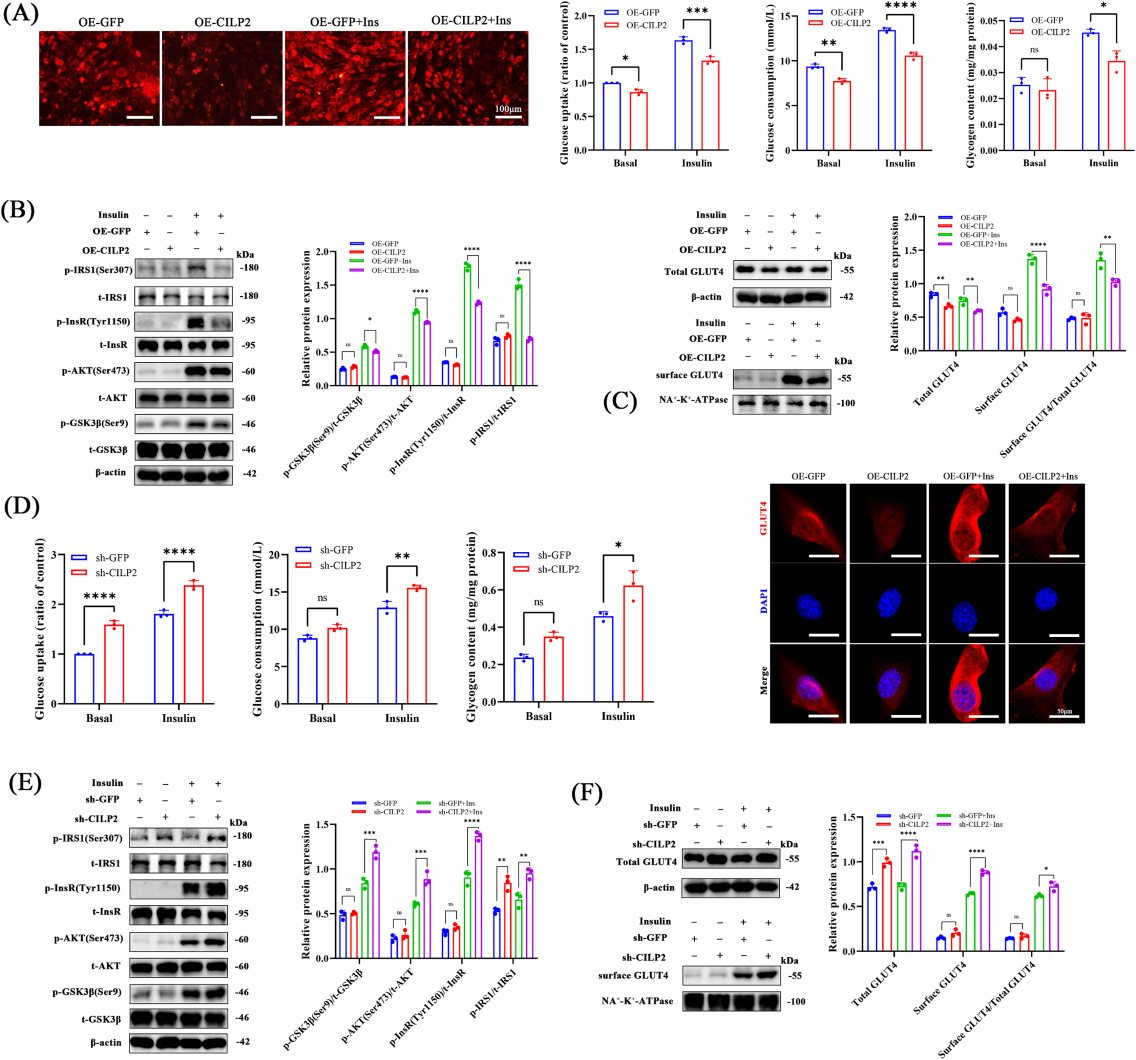
CILP2 is associated with glucose metabolism and insulin sensitivity in myotube. (A) Representative IF staining of glucose uptake and quantification of glucose uptake, glucose consumption and glycogen content in C2C12 of OE‐GFP and OE‐CILP2 without or with insulin, scale bars = 100 μm. (B) Western blotting and quantitative analysis of the levels of p‐GSK3β(Ser9), p‐AKT (Ser473), p‐InsR (Tyr1150) and p‐IRS1(Ser473) in C2C12 of OE‐GFP and OE‐CILP2 without or with insulin, *n* = 3. (C) Western blotting and quantitative analysis and representative IF staining of GLUT4 of the levels of total GLUT4, surface GLUT4 and surface GLUT4/total GLUT4 in C2C12 of OE‐GFP and OE‐CILP2 without or with insulin, *n* = 3. (D) Quantification of glucose uptake, glucose consumption and glycogen content in C2C12 of sh‐GFP and sh‐CILP2 without or with insulin, *n* = 3. (E) Western blotting and quantitative analysis of the levels of p‐GSK3β(Ser9), p‐AKT (Ser473), p‐InsR (Tyr1150) and p‐IRS1(Ser473) in C2C12 of sh‐GFP and sh‐CILP2 without or with insulin, *n* = 3. (F) Western blotting and quantitative analysis of the levels of total GLUT4, surface GLUT4 and surface GLUT4/total GLUT4 in C2C12 of sh‐GFP and sh‐CILP2 without or with insulin, *n* = 3. For all statistical plots, Values are shown as mean ± SD. Ns, no significance, *p < 0.05, **p < 0.01, ***p < 0.001, ****p < 0.0001. Statistical significance was determined by Student's *t* test (for A and D) or two‐way ANOVA (for B, C, E and F).

To determine the effect of CILP2 on glucose uptake in skeletal muscle, insulin was added to the differentiated myotubes following the overexpression or knockout of CILP2. With CILP2 overexpression, both basal and insulin‐stimulated glucose uptake were inhibited (Figure 5A), and glucose consumption in the medium supernatant was reduced. Additionally, OE‐CILP2 inhibited the insulin‐stimulated intracellular glycogen content (Figure 5A). Conversely, CILP2 knockout enhanced basal and insulin‐stimulated glucose uptake, glucose consumption and glycogen content (Figures S2A and 5D). Next, we examined changes in insulin signalling pathway proteins with and without insulin stimulation. Glucose uptake in skeletal muscle is driven primarily by an increase in insulin‐stimulated AKT signalling [31]. As shown in Figure 5B, OE‐CILP2 inhibited the phosphorylation of GSK3β, Akt, InsR and IRS1 under insulin stimulation. In turn, sh‐CILP2 not only promoted non‐insulin‐stimulated p‐IRS (Ser307) but also increased the expression of insulin‐stimulated p‐GSK3β (Ser9), p‐AKT (Ser473), p‐InsR (Tyr1150/1151) and p‐IRS (Ser307) (Figure 5E). Given that GLUT4 translocation to the membrane plays a central role in insulin stimulated glucose uptake in skeletal muscle [8], we next investigated whether CILP2 is involved in insulin‐stimulated GLUT4 mobilization. We observed that OE‐CILP2 significantly decreased GLUT4 translocation to the cell membrane, and IF also confirmed this phenomenon (Figure 5C). The sh‐CILP2 increased GLUT4 translocation (Figures 5F and S2B). Overall, these results suggest that CILP2 overexpression reduces insulin sensitivity and glucose metabolism in skeletal muscle.

### 3.7 CILP2 Promotes the Progression of Sarcopenia Through Regulating the Wnt/β‐Catenin Pathway

**FIGURE 6:**
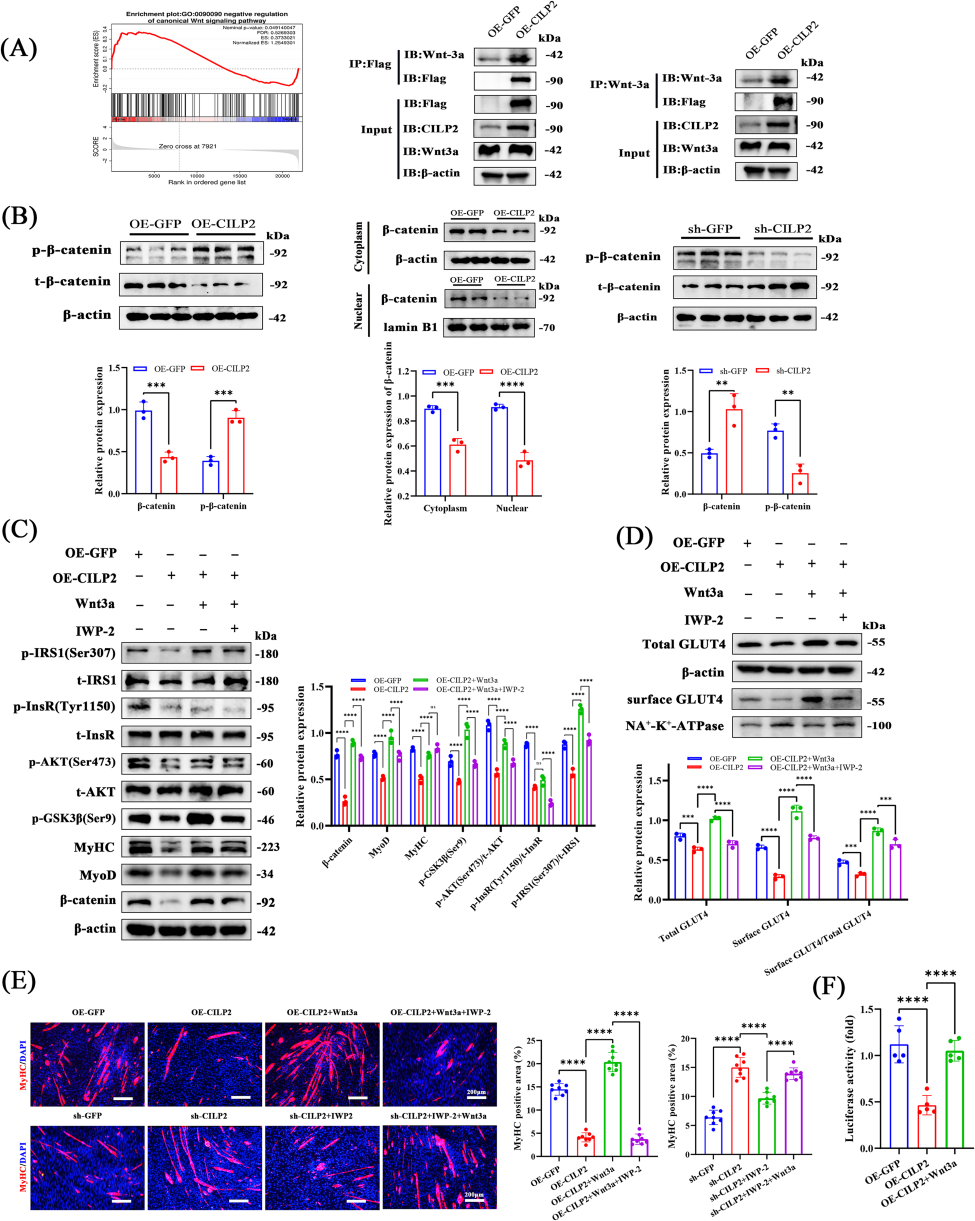
CILP2 promotes the progression of sarcopenia via the Wnt/beta‐catenin signalling pathway. (A) GSEA enrichment plot of DEGs related to canonical Wnt signalling pathway and the co‐immunoprecipitation experiments tested the interaction between CILP2 and Wnt3a in C2C12 myotubes. (B) Western blotting analysis revealed expression of p‐β‐catenin and β‐catenin and the levels of β‐catenin in cytoplasm and nuclear in OE‐GFP and OE‐CILP2 groups, *n* = 3. (C) Western blotting and quantitative analysis of the levels of β‐catenin, MyoD, MyHC, p‐GSK3β(Ser9), p‐AKT (Ser473), p‐InsR (Tyr1150) and p‐IRS1(Ser307) in C2C12 with OE‐CILP2, Wnt3a or IWP‐2 manipulation, *n* = 3. (D) Western blotting and quantitative analysis of the levels of total GLUT4, surface GLUT4 and surface GLUT4/total GLUT4 in C2C12 with OE‐CILP2, Wnt3a or IWP‐2 manipulation, *n* = 3. (E) Representative immunofluorescence staining of MyHC, scale bars = 200 μm. (F) TEF/LEF‐luciferase reporter activity of C2C12 in OE‐GFP, OE‐CILP2 and OE‐CILP2 with Wnt3a, *n* = 5. (E) *n* = 4, two fields per sample were selected. For all statistical plots, values are shown as mean ± SD. Ns, no significance, **p < 0.01, ***p < 0.001, ****p < 0.0001. Statistical significance was determined by Student's *t* test (for B) or one‐way ANOVA (for E and F) or two‐way ANOVA (for C and D).

We aimed to investigate the molecular signalling mechanisms that drive CILP2's regulation of myogenesis and glucose metabolism in skeletal muscle. We identified the gene expression profiles of OE‐GFP and OE‐CILP2 in myotubes using RNA‐Seq. Principal component analysis revealed that OE‐GFP and OE‐CILP2 strains were distinct (Figure S3A). A total of 559 DEGs were identified and visualized through volcano and heatmap (Figure S3B,C). GO enrichment analysis indicated that DEGs were significantly enriched in cellular processes, biological regulation, response to stimulus and metabolic processes (Figure S3D). According to the KEGG enrichment analysis, the significant pathways included glycolysis/gluconeogenesis, the PI3K‐Akt signalling pathway (related to protein synthesis) and the Wnt signalling pathway (Figure S3E). CILP2 is known to be closely associated with the Wnt/β‐catenin signalling pathway in neocortical development [32]. GSEA also suggested that the canonical Wnt signalling pathway was significantly linked to OE‐CILP2 (Figure 6A). We first predicted the molecular docking between CILP2 and Wnt3a using Z‐DOCK (Figure S3F) and found that CILP2 bound to Wnt3a at multiple sites via hydrogen bonds and salt bridges. The results of co‐immunoprecipitation (CO‐IP) experiments further indicated that FLAG‐tagged CILP2 pulled down the Wnt3a protein (Figure 6A). The accumulation of β‐catenin in the nucleus is a marker of canonical Wnt signal activation [33]. We subsequently found that an increase in p‐β‐catenin in OE‐CILP2 cells was accompanied by a decrease in β‐catenin. Meanwhile, OE‐CILP2 significantly reduced both cytoplasmic accumulation and nuclear transfer of β‐catenin (Figure 6B). In contrast, compared with that in the sh‐GFP group, the decrease in p‐β‐catenin in the sh‐CILP2 group was accompanied by an increase in β‐catenin (Figure 6B). Additionally, sh‐CILP2 markedly increased both cytoplasmic accumulation and nuclear transfer of β‐catenin (Figure S4A,B), suggesting that the degradation of β‐catenin was reduced and activated the Wnt/β‐catenin signalling pathway through a phosphorylation cascade.

Next, we treated OE‐CILP2 and sh‐CILP2 with the exogenous recombinant proteins Wnt3a and IWP2 (an inhibitor of Wnt protein). Notably, Wnt3a rescued the inhibition of myoblast proliferation, differentiation and insulin pathway‐associated proteins in OE‐CILP2 (Figure 6C). IF analysis revealed that Wnt3a significantly enhanced myotube formation in OE‐CILP2 (Figure 6E). Furthermore, Wnt3a restored GLUT4 translocation to the cell membrane (Figure 6D). IWP‐2 disrupted the promoting effects of Wnt3a, indicating that CILP2, similar to IWP‐2, also inhibited Wnt3a, impairing muscle regeneration, differentiation and glucose uptake. Conversely, IWP‐2 reversed the promotion of proliferation, differentiation and insulin pathway‐related proteins in sh‐CILP2 (Figure S4C,D). IWP‐2 reduced GLUT4 translocation to the cell membrane (Figure S4E,F). Importantly, Wnt3a can counteract the inhibitory effects caused by IWP‐2. Moreover, IWP‐2 also decreased the myotube fusion index in sh‐CILP2 (Figure 6E).

To further evaluate the activity of the Wnt pathway, we employed the TCF/LEF dual luciferase reporter assay system. We found that increased CILP2 in C2C12 could partially impair the Wnt/β‐catenin signalling pathway during myogenic differentiation. However, supplementation with Wnt3a restored the Wnt signalling pathway activity (Figure 6F). In summary, these results suggest that increased CILP2 in the in vitro model of muscle regeneration interacts with Wnt3a, thereby cascading the canonical Wnt signalling pathway during muscle development.

### 3.8 CILP2 Silencing Enhanced Endurance Exercise Capacity and Muscle Mass in SAMP8 Ageing Mice

**FIGURE 7:**
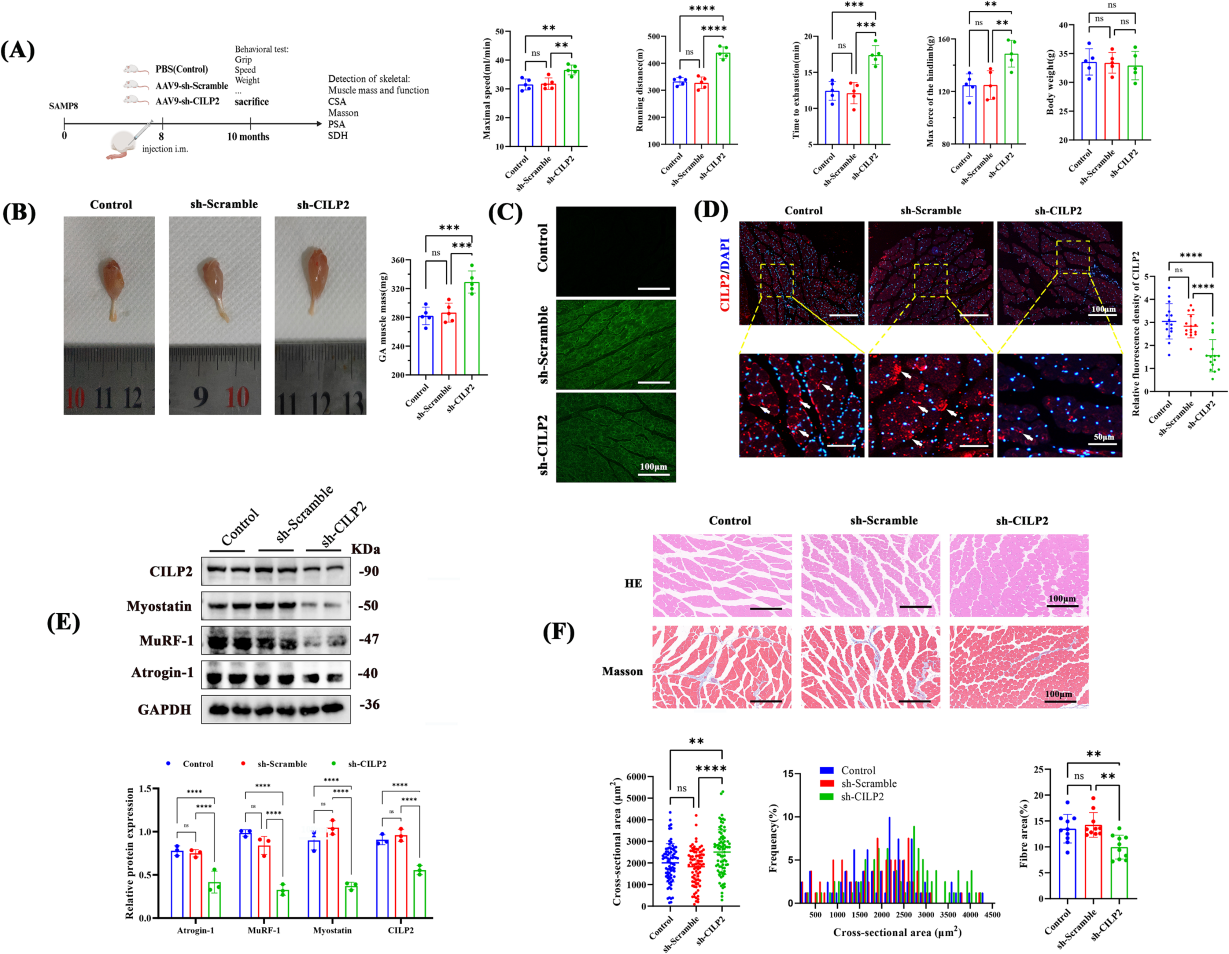
CILP2 knockout enhanced endurance exercise capacity and muscle mass in senescent SAMP8 mice. (A) Schematic diagram of animal experiments and physical performance assessment on a treadmill. After 2 months of treatment, measurements of maximal running speed, distance, running time to exhaustion, grip strength and body weight, *n* = 5. (B) Representative macro photographs of GAs of each group and quantitative analysis of GAs weight normalized to body weight, *n* = 5. (C) eGFP expression was visualized by fluorescence microscopy in frozen sections of the GA muscles of each group, scale bars = 100 μm. (D) Representative IF staining and the fluorescence density of CILP2 of each group, scale bars = 100 μm. (E) Western blotting and quantitative analysis detected the expression of CILP2, Myostatin, MuRF‐1 and Atrogin‐1 in GA muscle; GAPDH was used as a loading control, *n* = 3. (F) Representative HE staining (top) and Masson staining (bottom) for the GAs muscle of each group, and quantification, scale bars = 100 μm. (D) *n* = 5, three fields per sample were selected. (F) *n* = 5, 50 fibres per sample were selected. For all statistical plots, values are shown as mean ± SD. Ns, no significance, **p < 0.01, ***p < 0.001, ****p < 0.0001. Statistical significance was determined by one‐way ANOVA (for A, B, D and F) or two‐way ANOVA (for E). GA, gastrocnemius; HE, haematoxylin and eosin; CSA, cross‐sectional area. Control, mice injected with the same volume of PBS; AAV9‐sh‐Scramble, mice injected with scramble shRNA vector control; AAV9‐sh‐CILP2, mice injected with AAV9 vectors encoding sh‐CILP2.

We further conducted in vivo studies to explore whether CILP2 knockout promotes myogenic differentiation and glucose metabolism and alleviates sarcopenia. SAMP8 mice, as a model of accelerated ageing, have been extensively utilized in sarcopenia research. As illustrated in Figure 7A, compared to control and sh‐Scramble, sh‐CILP2 significantly improved maximal running speed, running distance, time to exhaustion and maximum force of the hindlimbs of 10‐month‐old SAMP8 mice. Additionally, AAV9‐sh‐CILP2 increased the volume and mass of the GA muscle (Figure 7B). Next, we prepared sections of the GA specimens for histological evaluation. In frozen sections, the high expression of eGFP in GA confirmed the in vivo transfection efficiency of the AAV9 vector (Figure 7C). IF (Figure 7D) and WB (Figure 7E) further confirmed that sh‐CILP2 significantly reduced CILP2 expression in GA muscle. Interestingly, sh‐CILP2 also significantly decreased the expression of muscular dystrophy proteins: Atrogin‐1, MuRF‐1 and Myostatin (Figure 7E). We observed that CILP2 knockout maintained a higher cross‐sectional area (CSA) of muscle fibres and reduced fibre area (Figure 7F).

### 3.9 CILP2 Ablation Improves Glucose Metabolism, Mitochondrial Oxidation and Regeneration Potential in the Skeletal Muscle of SAMP8 Ageing Mice via the Wnt/β‐Catenin Pathway

**FIGURE 8:**
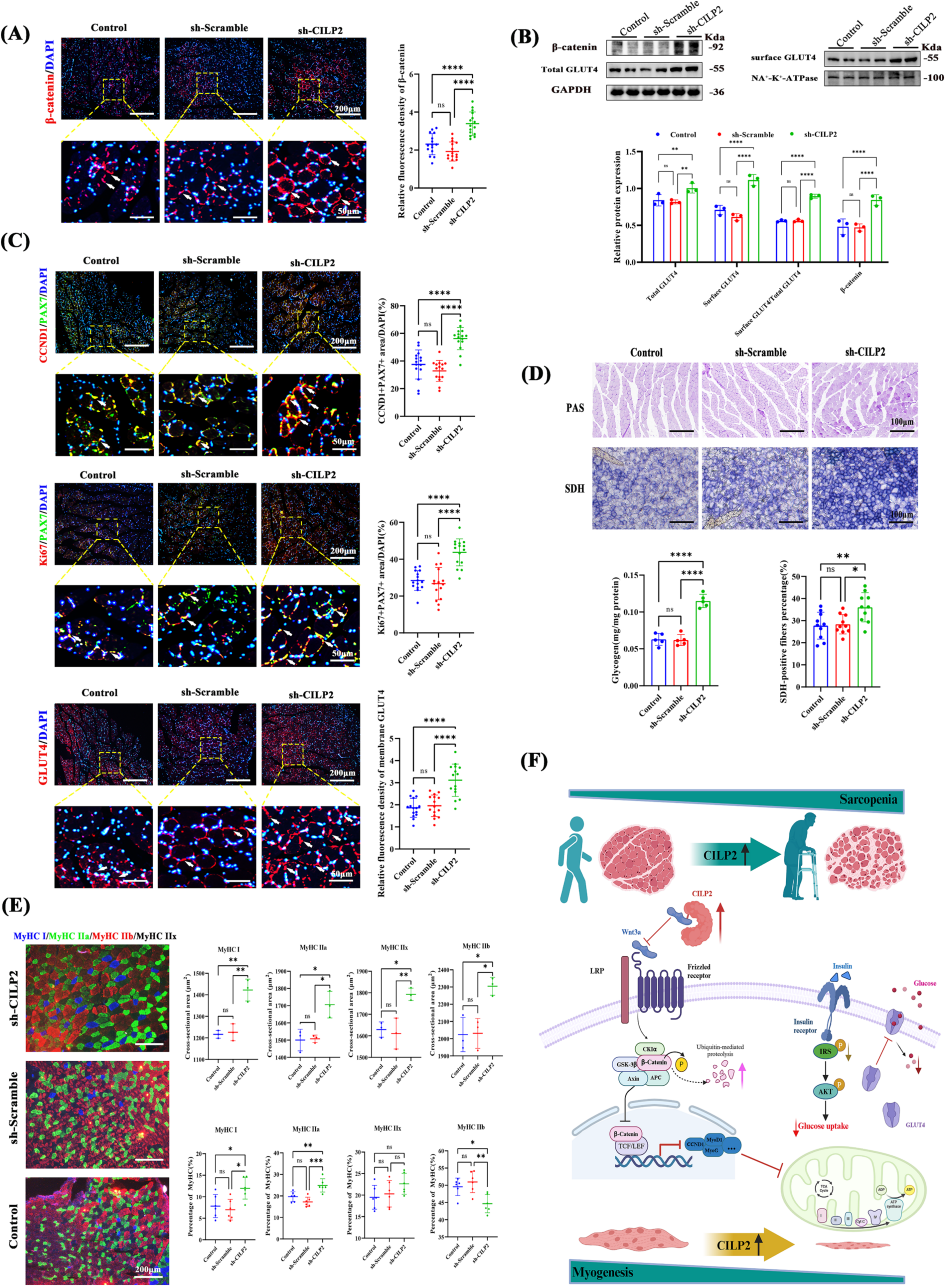
CILP2 knockout improves skeletal muscle regeneration and glucose metabolism potential in aging SAMP8 mice via the Wnt/β‐catenin pathway. (A) Representative IF staining and the fluorescence density of β‐catenin of GAs muscle in each group, scale bars = 200 μm. (B) Western blotting analysis revealed expression of β‐catenin, GLUT4 protein and surface GLUT4 and quantification, *n* = 3. (C) Representative IF staining for overlapping fluorescence of CCND1 or Ki67 and PAX7 and GLUT4 in GAs from each group, and quantification, scale bars = 200 μm. (D) Representative PAS staining (top) and SDH staining (bottom) in GAs muscle of each group, and quantification of glycogen content, scale bars = 100 μm. (E) Representative IF staining of slow MyHC (top) or fast MyHC (bottom) and Laminin and quantitative analysis of the percentage of slow MyHC or fast MyHC of GAs muscle of each group, scale bars = 200 μm. (F) Schematic summary of the regulatory role of CILP2 in myogenic differentiation. (A and C) *n* = 5, three fields per sample were selected. (D) *n* = 5, two fields per sample were selected. (E) *n* = 3, mean value of 10 fibres per sample were selected in CSA, and two fields per sample were selected in percentage. For all statistical plots, values are shown as mean ± SD. Ns, no significance, *p < 0.05, **p < 0.01, ****p < 0.0001. Statistical significance was determined by one‐way ANOVA (for A, C, D and E) or two‐way ANOVA (for B). GA, gastrocnemius; PAS, Periodic Acid‐Schiff. Control, mice injected with the same volume of PBS; AAV9‐sh‐Scramble, mice injected with scramble shRNA vector control; AAV9‐sh‐CILP2, mice injected with AAV9 vectors encoding sh‐CILP2.

To determine whether CILP2 knockdown can enhance the Wnt/β‐catenin signalling pathway, we conducted IF staining and WB for the target gene transcription factor CCND1 of β‐catenin and the Wnt signalling pathway. Compared with control and AAV9‐sh‐Scramble, AAV9‐sh‐CILP2 significantly elevated the expression of β‐catenin (Figure 8A,B) and CCND1 (Figure 8C). Additionally, IF co‐staining of PAX7 and Ki67 demonstrated that CILP2 ablation stimulated the proliferation of muscle satellite cells in SAMP8 mice (Figure 8C). GLUT4 is the primary receptor for glucose uptake in skeletal muscle [34], and WB analysis revealed that sh‐CILP2 facilitated GLUT4 translocation to the cell membrane (Figure 8B). IF staining also indicated that GLUT4 expression in the membrane was significantly elevated in the sh‐CILP2 group (Figure 8C). To evaluate the status of glucose metabolism in vivo, we analysed glycogen synthesis and muscle fibre characteristics associated with oxidation using PAS staining, glycogen assays and SDH staining (Figure 8D). The results showed that sh‐CILP2 significantly increased glycogen content and the accumulation of metabolically active SDH‐positive fibres. Furthermore, IF analysis revealed that the mean CSA of MyHC I, IIa, IIx and IIb muscle fibres, as well as the percentage of I and IIa muscle fibres in the AAV9‐sh‐CILP2 group, were significantly higher than those in the Control and AAV9‐sh‐Scramble groups (Figure 8E). There was no significant change in the proportion of MyHC IIx fibres across the three groups. Notably, oxidative fibres (Type I and Type IIa) contain more mitochondria and are primarily regulated by the metabolic pattern of oxidative phosphorylation [S1]. In summary, these findings suggest that sh‐CILP2 prevents muscle atrophy by activating the Wnt/β‐catenin signalling pathway to enhance glucose uptake and glycogen synthesis, improve the oxidative capacity of muscle fibres and promote skeletal muscle regeneration.

## 4 Discussion

Low muscle mass or strength can result in negative outcomes such as hip fractures, disability and diminished quality of life in older adults [S2, S3]. Although prevalent among the elderly, the underlying molecular mechanisms are still being explored [S4]. Skeletal muscle, the primary organ involved in glucose clearance, is closely associated with sarcopenia, glucose metabolism and IR [S5]. Enhancing muscle glucose metabolism presents a novel and effective strategy for addressing sarcopenia. The findings of this study, depicted in Figure 8F, underscore the vital role of the CILP2‐mediated Wnt/β‐catenin axis in myogenesis and glucose metabolism. Consequently, inhibiting CILP2 signalling enhances glucose metabolism and mitochondrial function, making it a potential therapeutic target for sarcopenia.

This study demonstrates a significant association between elevated CILP2 levels and reduced glycogen synthesis and protein degradation in a muscular dystrophy model, as observed in patients with sarcopenia and aged mice. The proliferation of myogenic progenitor cells is regarded as the initial stage involved in skeletal muscle development [S6, S7]. Consistent with previous findings [S8, S9], changes in the expression of PCNA and Ki67 reflect the proliferative capacity of cells. Combined, the results of the CCK8 and EDU assays show that overexpression or knockdown of KERA leads to decreased or increased proliferation of C2C12 myoblasts. It is well established that myogenic regulatory factors (MRFs), such as MyoD1 and MyoG, are key transcription factors in myogenic differentiation and drive the formation of multinucleated myotubes. MyHC is recognized as a terminal marker of myogenic differentiation [S8, S9]. The overexpression or knockdown of CILP2 led to decreased or increased expression of MRFs, which also influenced differentiation potential. In addition to the myostatin pathway impairing skeletal muscle development [S10], an imbalance between muscle protein synthesis and degradation during ageing also contributes to a net loss of muscle mass. Muscle protein degradation is mediated primarily by the ubiquitin‐proteasome system, with the muscle‐specific E3 ubiquitin‐ligase enzymes Atrogin‐1 and MuRF‐1 upregulated during ageing [S11, S12]. CILP2 knockdown inhibited myostatin and muscle protein degradation in both C2C12 myotubes and SAMP8 ageing mice. Collectively, these findings suggest that CILP2 overexpression inhibits the process of myogenic differentiation.

Skeletal muscle serves as the primary organ involved in insulin‐induced glucose metabolism. The loss of muscle mass during ageing is closely linked to IR and disorders in glucose metabolism [S13]. Up to 28% of diabetic patients over the age of 50 experience sarcopenia [S14]. However, the molecular mechanisms that contribute to increased IR in ageing muscle remain unclear. Massart et al. demonstrated that aerobic exercise training can enhance skeletal muscle function by boosting insulin signalling and glucose uptake [S15]. Prabakaran et al. found that hormone therapy can increase mitochondrial abundance and improve glucose metabolism, thereby mitigating mass loss associated with ageing‐related sarcopenia [S15]. Yang et al. showed that microRNA‐92b in skeletal muscle regulates exercise capacity by influencing glucose metabolism [S16]. Guo et al. reported that PGC‐1α4 enhances insulin signalling and promotes glucose uptake in skeletal muscle, presenting a potential strategy for addressing age‐related diseases and sarcopenia [S17]. Insulin has also been shown to stimulate muscle protein synthesis [S18]. In C2C12 myoblasts, CILP2 knockdown boosts insulin‐induced glucose uptake and GLUT4 translocation. Similarly, CILP2 knockdown increases glycogen synthesis and mitochondrial metabolism in ageing mouse skeletal muscle. This outcome parallels exercise training, which improves insulin sensitivity and mitochondrial biosynthesis in skeletal muscle [S19]. Given that dysglycaemia is associated with a significant rise in serum CILP2 in patients [S20], regulating CILP2 expression may also influence systemic glucose metabolism. Enhancing GLUT4 translocation and the phosphorylation of insulin IRS‐1 and Akt are also therapeutic targets in obesity and diabetic sarcopenia [S21]. Additionally, skeletal muscle experiences changes in fibre type composition as it ages [S22]. The change in fibre type following CILP2 knockdown differs from the transition of type II (fast‐twitch) myofibers to type I (slow‐twitch) myofibers. Instead, it promoted a regeneration primarily characterized by oxidative myofibers (type I and type IIa) alongside a slower regeneration of glycolytic myofibers (type IIx and type IIb), indicating that CILP2 is involved in the oxidative phosphorylation of muscle cells. Consistent with previous findings [S23], the increase in skeletal muscle oxidative phenotype also enhanced the endurance capacity of mice, which was further confirmed through exhaustion tests on the treadmill. However, additional research is necessary to ascertain whether CILP2 influences changes in glycolysis and metabolism in skeletal muscle.

Most researchers have confirmed that the Wnt signalling pathway is linked to the ageing of muscle stem cells and the transition from a myogenic lineage to a fibrogenic lineage [S24, S25]. As an evolutionarily conserved pathway, substantial evidence indicates that increases in Wnt signalling can delay age‐related deficits [S26]. Integrating previous research findings, Wnt signalling not only stimulates myogenic differentiation but also influences the loss of myogenic cell fate [S27–S30]. For instance, through moderate activation of the Wnt signalling pathway, miR‐145‐5p mimics the significant myogenic differentiation potential of human foetal cartilage‐derived progenitor cells [S31]. Wang et al. reported that the Wnt/β‐catenin signalling pathway serves as a primary regulatory mechanism for low‐magnitude high‐frequency vibration and β‐hydroxy‐β‐methylbutyrate combined therapy to enhance muscle strength [S32]. Yin et al. also found that Plectin activates canonical Wnt signalling‐mediated skeletal muscle regeneration by stabilizing Dishevelled‐2 and downregulating the autophagy degradation system [S33]. β‐catenin is a crucial participant in the signal transduction pathway of Wnt signalling during muscle development. Functionally, we found that CILP2, as one of the key regulatory mediators of Wnt signalling, can interact with Wnt3a to inactivate the canonical Wnt signalling and inhibit the nuclear translocation of β‐catenin. This process may impact several important MRFs such as MyoD1. Therefore, silencing CILP2 promotes the proliferation and pro‐differentiating properties of skeletal muscle and C2C12 myoblasts. We hypothesize that the primary reason is to restore impaired IR and mitochondrial function through the Wnt/β‐catenin pathway, thereby enhancing glucose uptake and metabolism. This aligns with previous research suggesting that CILP2 is associated with metabolic syndrome [S34].

Due to its association with macrophage‐related subtypes, CILP2 can serve as a biomarker for prognosis across various cancers and as a potential target for immunotherapy [S35, S36]. Furthermore, CILP2 plays a role in the progression of fibrotic diseases through its interaction with ATP citrate lyase [S37]. Notably, our groundbreaking discovery indicates that CILP2 is a promising biomarker for sarcopenia. Our findings expand understanding of the mechanisms underlying glucose metabolism in sarcopenia. However, several limitations should be acknowledged. First, the upstream mechanisms regulating the abnormal expression of CILP2 remain unclear. Second, CILP2 may exert multi‐target effects in skeletal muscle, necessitating further validation to ascertain whether its role in regulating myogenic differentiation and glucose metabolism is critical. Third, we did not validate our conclusions in natural ageing and other accelerated ageing mouse models of sarcopenia (SAMP10) [S38]. Lastly, we have not developed muscle‐specific CILP2 knockout mice, which limits our ability to thoroughly investigate the function of CILP2 in muscle ageing.

In summary, our results illustrate the advantages of silencing CILP2 on glucose metabolism, muscle fibre size and running distance in age‐related muscle atrophy. Further studies have revealed that CILP2 may inhibit glucose uptake and utilization by mediating insulin sensitivity and GLUT4 translocation via the Wnt/β‐catenin signalling pathway. This study identified CILP2 as a potential biological target for glucose metabolism in the treatment of sarcopenia, which should be prioritized in future research.

## Ethics Statement

All animal experiments were approved by the Animal Ethics Committee of Fujian Provincial Hospital (IACUC‐FPH‐SL‐20240228[0121]). Besides, this study was performed in line with the principles of the Declaration of Helsinki. Approval was granted by the Ethics Committee of Fujian Provincial Hospital (No. K2023‐06‐007). Informed consent was obtained from individual participants and/or their legal guardians in the study. Besides, the accessed patient data complied with relevant protection, privacy guidelines and regulations.

## Conflicts of Interest

The authors declare no conflicts of interest.

## Supporting information


**Figure S1** Muscle atrophy was evident in patients with sarcopenia and in mice aged 24 months, and glucose metabolism in skeletal muscle is decreased in sarcopenia and aged mice. (A) Representative HE staining and quantitative analysis of CSA of quadriceps in non‐sarcopenia (left) and sarcopenia (right), scale bars = 100 μm. (B) Representative Masson staining and quantitative analysis of the fibre area of quadriceps in non‐sarcopenia (left) and sarcopenia (right), scale bars = 100 μm. (C) Representative PAS staining examined the glycogen level in non‐sarcopenia group (left) and sarcopenia group (right), scale bars = 100 μm. (D) Glycogen content of quadriceps muscle was quantitatively detected using Glycogen Assay Kit, *n* = 3. (E) Representative IF staining of fast MyHC (top) or slow MyHC (bottom) and quantitative analysis of the percentage of fast MyHC or slow MyHC of quadriceps muscle in the non‐sarcopenia and sarcopenia groups, scale bars = 100 μm. (F) Representative HE staining and quantitative analysis of CSA of GAs in 3‐ (left) and 24‐month (right) mice groups, scale bars = 100 μm. (G) Representative Masson staining and quantitative analysis of the fibre area of quadriceps in 3‐ (left) and 24‐month (right) mice groups, scale bars = 100 μm. (H) Representative PAS staining examined the glycogen level in GAs muscle of 3‐month group (left) and 24‐month group (right), scale bars = 100 μm. (I) Glycogen content of GAs muscle was quantitatively detected, *n* = 3. (J) Representative IF staining of fast MyHC (top) or slow MyHC (bottom) and Laminin and quantitative analysis of the percentage of fast MyHC or slow MyHC of GAs muscle in the 3‐ and 24‐month‐old mice groups, scale bars = 100 μm. (A and F) *n* = 5, 50 fibres per sample were selected. (B and G) *n* = 5, two fields per sample were selected. (E and J) *n* = 5, four fields per sample were selected. For all statistical plots, values are shown as mean ± SD, **p < 0.01, ***p < 0.001, ****p < 0.0001. Statistical significance was determined by Student's *t* test. GA, gastrocnemius; HE, haematoxylin and eosin; CSA, cross‐sectional area; PAS, Periodic Acid‐Schiff.

**Figure S2** CILP2 knockout promotes glucose uptake and GLUT4 translocation. (A) Representative IF staining of glucose uptake in C2C12 of shE‐GFP and sh‐CILP2 without or with insulin, scale bars = 100 μm. (B) Representative IF staining of GLUT4 in C2C12 of sh‐GFP and sh‐CILP2 without or with insulin, scale bars = 50 μm.

**Figure S3** Transcriptomic analysis of OE‐GFP and OE‐CILP2. (A) Principal component analysis (PCA) of the RNA‐sequencing (RNA‐Seq) data from C2C12 cells in OE‐GFP and OE‐CILP2 groups. (B) The volcano plot of the RNA‐Seq data. The red and blue data points represent the upregulated (log2fold change > 1, *p* < 0.05) and downregulated (log2 fold change < −1, *p* < 0.05) genes, respectively. (C) Heatmap showing differentially expressed genes (DEGs). (D) Gene ontology analysis of the DEGs. (E) KEGG enrichment analysis of the DEGs. (F) Molecular docking between CILP2 and Wnt3a. The red matrix represents focus content.

**Figure S4** CILP2 knockout improves insulin sensitivity and glucose uptake by enhancing the Wnt/ beta‐catenin pathway. (A and B) Western blotting and quantitative analysis of the levels of β‐catenin in cytoplasm and nuclear of sh‐GFP and sh‐CILP2 groups, *n* = 3. (C and D) Western blotting and quantitative analysis of the levels of β‐catenin, MyoD, MyHC, p‐GSK3β(Ser9), p‐AKT(Ser473), p‐InsR(Tyr1150) and p‐IRS1(Ser307) in C2C12 with sh‐CILP2, IWP‐2 or Wnt3a manipulation, *n* = 3. (E and F) Western blotting and quantitative analysis of the levels of total GLUT4, surface GLUT4 and surface GLUT4/total GLUT4 in C2C12 with sh‐CILP2, IWP‐2, or Wnt3a manipulation, *n* = 3. For all statistical plots, values are shown as mean ± SD. *p < 0.05, **p < 0.01, ***p < 0.001, ****p < 0.0001. Statistical significance was determined by Student's *t* test (for A) or one‐way ANOVA (for F) or two‐way ANOVA (for D).

**Table S1** The sequences of sh‐RNAs
**Table S2.** Primers used in RT‐qPCR experiments
**Table S3.** Antibodies and their application
**Table S4.** Clinical characteristics of patients with or without sarcopenia

## Data Availability

The paper and the supporting information present all data needed to evaluate the conclusions. Datasets used and/or analysed during the current study are available from the corresponding author on reasonable request.
