# Supplementary figures and images for: Inhibition of CILP2 Improves Glucose Metabolism and Mitochondrial Dysfunction in Sarcopenia via the Wnt Signalling Pathway
Source: J Cachexia Sarcopenia Muscle. 2024 Oct 10;15(6):2544–58. doi: 10.1002/jcsm.13597 (PMC11634484; doi:10.1002/jcsm.13597)

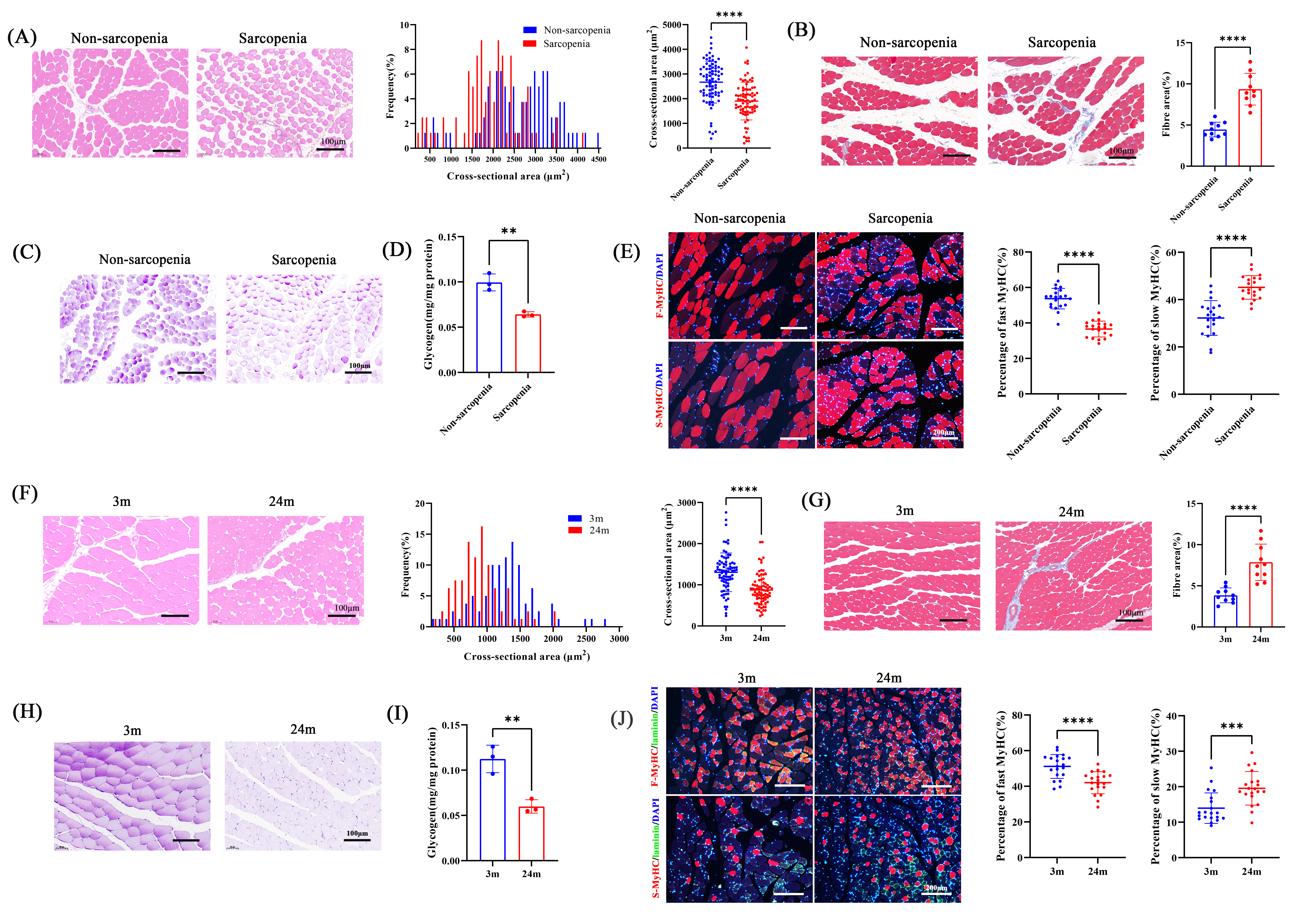

Supplement: Supplementary file 1 — Figure S1 Muscle atrophy was evident in patients with sarcopenia and in mice aged 24 months, and glucose metabolism in skeletal muscle is decreased in sarcopenia and aged mice. (A) Representative HE staining and quantitative analysis of CSA of quadriceps in non‐sarcopenia (left) and sarcopenia (right), scale bars = 100 μm. (B) Representative Masson staining and quantitative analysis of the fibre area of quadriceps in non‐sarcopenia (left) and sarcopenia (right), scale bars = 100 μm. (C) Representative PAS staining examined the glycogen level in non‐sarcopenia group (left) and sarcopenia group (right), scale bars = 100 μm. (D) Glycogen content of quadriceps muscle was quantitatively detected using Glycogen Assay Kit, n = 3. (E) Representative IF staining of fast MyHC (top) or slow MyHC (bottom) and quantitative analysis of the percentage of fast MyHC or slow MyHC of quadriceps muscle in the non‐sarcopenia and sarcopenia groups, scale bars = 100 μm. (F) Representative HE staining and quantitative analysis of CSA of GAs in 3‐ (left) and 24‐month (right) mice groups, scale bars = 100 μm. (G) Representative Masson staining and quantitative analysis of the fibre area of quadriceps in 3‐ (left) and 24‐month (right) mice groups, scale bars = 100 μm. (H) Representative PAS staining examined the glycogen level in GAs muscle of 3‐month group (left) and 24‐month group (right), scale bars = 100 μm. (I) Glycogen content of GAs muscle was quantitatively detected, n = 3. (J) Representative IF staining of fast MyHC (top) or slow MyHC (bottom) and Laminin and quantitative analysis of the percentage of fast MyHC or slow MyHC of GAs muscle in the 3‐ and 24‐month‐old mice groups, scale bars = 100 μm. (A and F) n = 5, 50 fibres per sample were selected. (B and G) n = 5, two fields per sample were selected. (E and J) n = 5, four fields per sample were selected. For all statistical plots, values are shown as mean ± SD, **p < 0.01, ***p < 0.001, ****p < 0.0001. Statistical significance was [file JCSM-15-2544-s004.png]

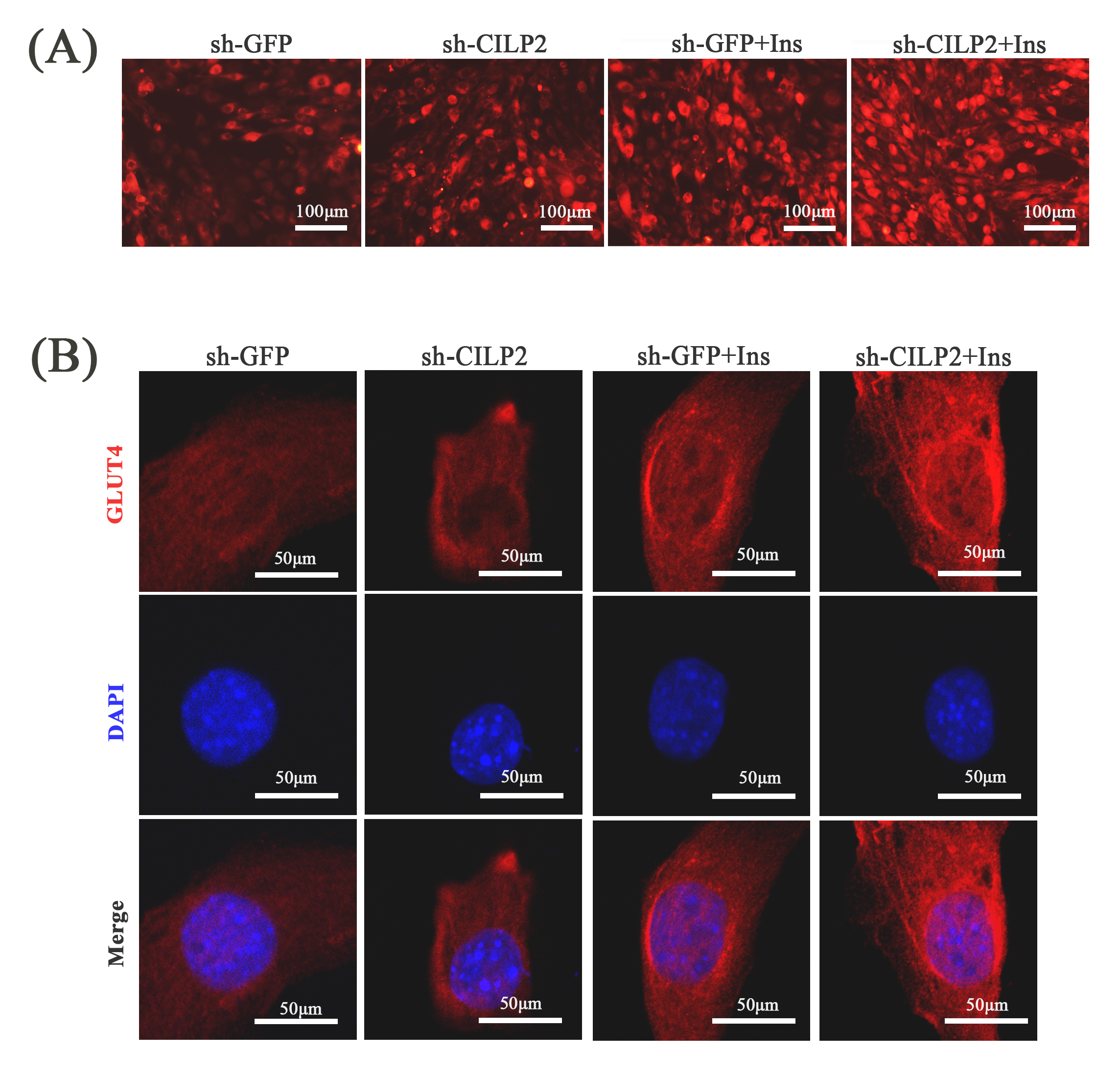

Supplement: Supplementary file 2 — Figure S2 CILP2 knockout promotes glucose uptake and GLUT4 translocation. (A) Representative IF staining of glucose uptake in C2C12 of shE‐GFP and sh‐CILP2 without or with insulin, scale bars = 100 μm. (B) Representative IF staining of GLUT4 in C2C12 of sh‐GFP and sh‐CILP2 without or with insulin, scale bars = 50 μm. [file JCSM-15-2544-s003.png]

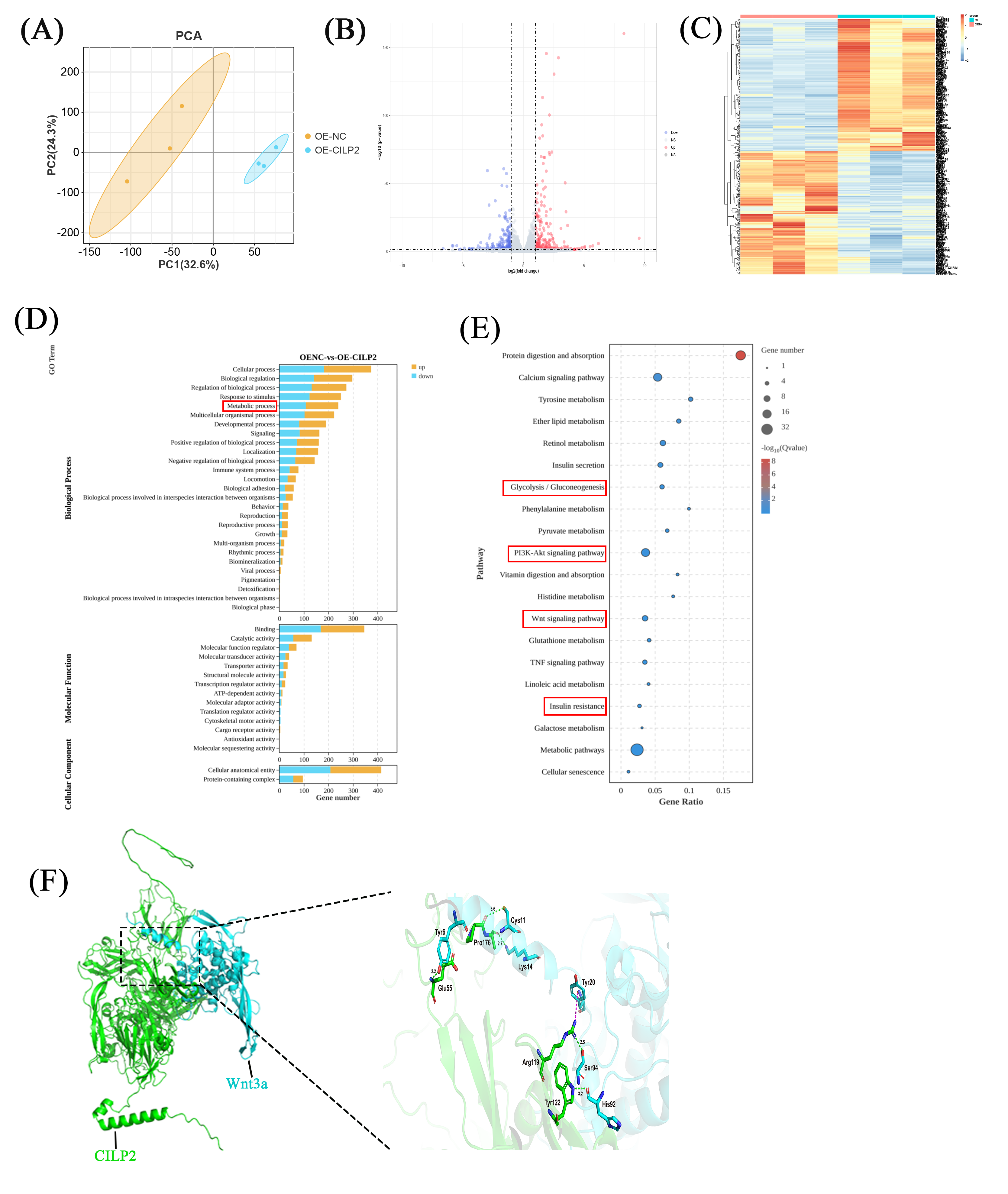

Supplement: Supplementary file 3 — Figure S3 Transcriptomic analysis of OE‐GFP and OE‐CILP2. (A) Principal component analysis (PCA) of the RNA‐sequencing (RNA‐Seq) data from C2C12 cells in OE‐GFP and OE‐CILP2 groups. (B) The volcano plot of the RNA‐Seq data. The red and blue data points represent the upregulated (log2fold change > 1, p < 0.05) and downregulated (log2 fold change < −1, p < 0.05) genes, respectively. (C) Heatmap showing differentially expressed genes (DEGs). (D) Gene ontology analysis of the DEGs. (E) KEGG enrichment analysis of the DEGs. (F) Molecular docking between CILP2 and Wnt3a. The red matrix represents focus content. [file JCSM-15-2544-s005.png]

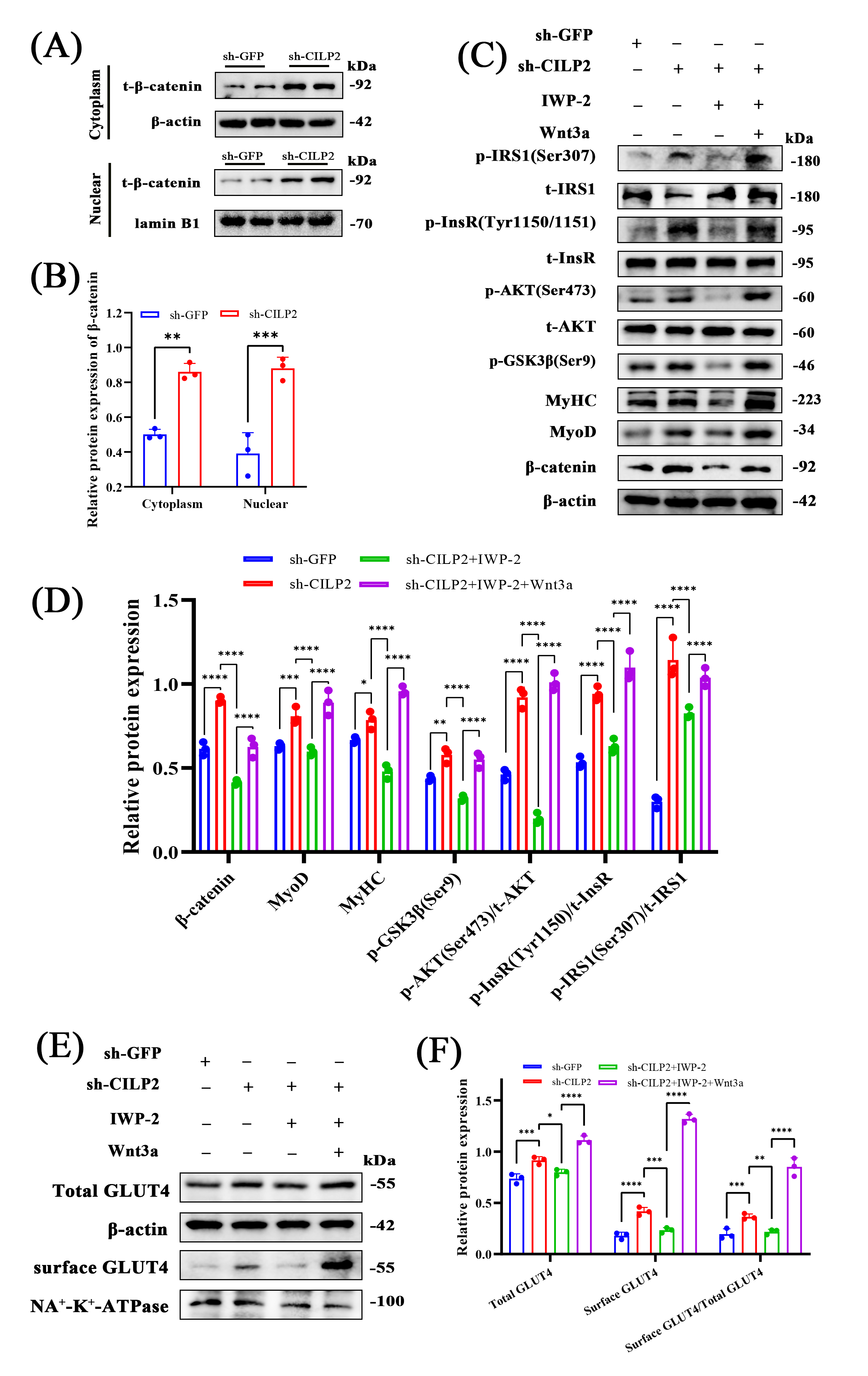

Supplement: Supplementary file 4 — Figure S4 CILP2 knockout improves insulin sensitivity and glucose uptake by enhancing the Wnt/ beta‐catenin pathway. (A and B) Western blotting and quantitative analysis of the levels of β‐catenin in cytoplasm and nuclear of sh‐GFP and sh‐CILP2 groups, n = 3. (C and D) Western blotting and quantitative analysis of the levels of β‐catenin, MyoD, MyHC, p‐GSK3β(Ser9), p‐AKT(Ser473), p‐InsR(Tyr1150) and p‐IRS1(Ser307) in C2C12 with sh‐CILP2, IWP‐2 or Wnt3a manipulation, n = 3. (E and F) Western blotting and quantitative analysis of the levels of total GLUT4, surface GLUT4 and surface GLUT4/total GLUT4 in C2C12 with sh‐CILP2, IWP‐2, or Wnt3a manipulation, n = 3. For all statistical plots, values are shown as mean ± SD. *p < 0.05, **p < 0.01, ***p < 0.001, ****p < 0.0001. Statistical significance was determined by Student's t test (for A) or one‐way ANOVA (for F) or two‐way ANOVA (for D). [file JCSM-15-2544-s002.png]
