# Supplementary material for: Inhibition of CILP2 Improves Glucose Metabolism and Mitochondrial Dysfunction in Sarcopenia via the Wnt Signalling Pathway
Source: J Cachexia Sarcopenia Muscle. 2024 Oct 10;15(6):2544–58. doi: 10.1002/jcsm.13597 (PMC11634484; doi:10.1002/jcsm.13597)
Supplement: Supplementary file 5 — Table S1 The sequences of sh‐RNAs Table S2. Primers used in RT‐qPCR experiments Table S3. Antibodies and their application Table S4. Clinical characteristics of patients with or without sarcopenia [file JCSM-15-2544-s001.docx]

**Supplemental** **Figure 1. Muscle atrophy was evident in patients with sarcopenia and in mice aged 24 months, and glucose metabolism in skeletal muscle is decreased in sarcopenia and aged mice.** (A) Representative HE staining and quantitative analysis of CSA of quadriceps in non-sarcopenia (left) and sarcopenia (right), scale bars=100μm. (B) Representative Masson staining and quantitative analysis of the fiber area of quadriceps in non-sarcopenia (left) and sarcopenia (right), scale bars=100μm. (C) Representative PAS staining examined the glycogen level in non-sarcopenia group (left) and sarcopenia group (right), scale bars=100μm. (D) Glycogen content of quadriceps muscle was quantitatively detected using Glycogen Assay Kit, n = 3. (E) Representative IF staining of fast MyHC (top) or slow MyHC (bottom), and quantitative analysis of the percentage of fast MyHC or slow MyHC of quadriceps muscle in the non-sarcopenia and sarcopenia groups, scale bars=100μm. (F) Representative HE staining and quantitative analysis of CSA of GAs in 3m (left) and 24m (right) mice groups, scale bars=100μm. (G) Representative Masson staining and quantitative analysis of the fiber area of quadriceps in 3m (left) and 24m (right) mice groups, scale bars=100μm. (H) Representative PAS staining examined the glycogen level in GAs muscle of 3m group (left) and 24m group (right), scale bars=100μm. (I) Glycogen content of GAs muscle was quantitatively detected, n=3. (J) Representative IF staining of fast MyHC (top) or slow MyHC (bottom) and Laminin, and quantitative analysis of the percentage of fast MyHC or slow MyHC of GAs muscle in the 3m and 24m old mice groups, scale bars=100μm. (A and F) n =5, 50 fibers per sample were selected. (B and G) n =5, two fields per sample were selected. (E and J) n =5, four fields per sample were selected. For all statistical plots, Values are shown as mean ± SD, **p < 0.01, ***p < 0.001, ****p < 0.0001. Statistical significance was determined by Student's t test. GA, gastrocnemius; HE, haematoxylin and eosin; CSA, cross-sectional area; PAS, Periodic Acid-Schiff.

**Supplemental** **Figure 2. CILP2 knockout promotes glucose uptake and GLUT4 translocation.** (A) Representative IF staining of glucose uptake in C2C12 of shE-GFP and sh-CILP2 without or with insulin, scale bars=100μm. (B) Representative IF staining of GLUT4 in C2C12 of sh-GFP and sh-CILP2 without or with insulin, scale bars=50μm.

**Supplemental** **Figure 3. Transcriptomic analysis of OE-GFP and OE-CILP2.** (A) Principal component analysis (PCA) of the RNA‐sequencing (RNA‐Seq) data from C2C12 cells in OE-GFP and OE-CILP2 groups. (B) The volcano plot of the RNA‐Seq data. The red and blue data points represent the up‐regulated (log2fold change > 1, P < 0.05) and down‐regulated (log2 fold change < −1, P < 0.05) genes, respectively. (C) Heatmap showing differentially expressed genes (DEGs). (D) Gene ontology analysis of the DEGs. (E) KEGG enrichment analysis of the DEGs. (F) Molecular docking between CILP2 and Wnt3a. The red matrix represents focus content.

**Supplemental** **Figure 4. CILP2 knockout improves insulin sensitivity and glucose uptake by enhancing the Wnt/ beta-catenin pathway.** (A-B) Western blotting and quantitative analysis of the levels of β-catenin in cytoplasm and nuclear of sh-GFP and sh-CILP2 groups, n = 3. (C-D) Western blotting and quantitative analysis of the levels of β-catenin, MyoD, MyHC, p-GSK3β(Ser9), p-AKT(Ser473), p-InsR(Tyr1150) and p-IRS1(Ser307) in C2C12 with sh-CILP2, IWP-2, or Wnt3a manipulation, n=3. (E-F) Western blotting and quantitative analysis of the levels of total GLUT4, surface GLUT4 and surface GLUT4/total GLUT4 in C2C12 with sh-CILP2, IWP-2, or Wnt3a manipulation, n=3. For all statistical plots, Values are shown as mean ± SD. *p < 0.05, **p < 0.01, ***p < 0.001, ****p < 0.0001. Statistical significance was determined by Student's t test (for A) or one‐way ANOVA (for F) or two‐way ANOVA (for D).

| **Table S1. The sequences of sh-RNAs** | |
| --- | --- |
| Name | Sequence |
| sh-CILP2 | 5′-GCCACAACTATGGCGTCTACA-3′ |
| Sh-NC | 5′-TTCTCCGAACGTGTCACGT-3′ |
| AAV9-sh-CILP2 | 5′-GCCACAACTATGGCGTCTACA-3′ |
| AAV9-sh-Scramble | 5′-CGCTGAGTACTTCGAAATGTC-3′ |

| **Table S2. Primers used in RT-qPCR experiments** | | |
| --- | --- | --- |
| Name | Forward primer | Reverse primer |
| CILP2 (mus) | 5′-TGTCAGCGCCCAGATGAATG-3′ | 5′-AGGTACGGCTTCCCTAACTC-3′ |
| CILP2 (homo) | 5′-ACGGACTCCTTCGGGATTAC-3′ | 5′-CAGTGTAGACGCCATAGTTGTG-3′ |
| GAPDH (mus) | 5′-TGGAAAGCTGTGGCGTGATG-3′ | 5′-TACTTGGCAGGTTTCTCCAGG-3′ |
| GAPDH (homo) | 5′-GGTGTGAACCATGAGAAGTATGA-3′ | 5′-GAGTCCTTCCACGATACCAAAG-3′ |

| **Table S3. Antibodies and their application** | | | | |
| --- | --- | --- | --- | --- |
| Antibody name | Catalogue Number | Source | Dilution ratio | |
|  |  |  | WB | IF |
| Anti-GAPDH antibody | HRP-60004 | Proteintech | 1/10000 |  |
| Anti-MyoD1 antibody | ab203383 | Abcam | 1/100 |  |
| Anti-MyoG antibody | ab124800 | Abcam | 1/200 | 1/500 |
| Anti-Fast Myosin Skeletal Heavy chain antibody | ab91506 | Abcam | 1/1000 | 1/1000 |
| Anti-Ki67 antibody | A16919 | ABclonal | 1/1000 | 1/200 |
| Anti-MuRF-1 antibody | A3101 | ABclonal | 1/2000 |  |
| Anti-Atrogin-1 antibody | A3193 | ABclonal | 1/2000 |  |
| Anti-Myostatin antibody | A22725 | ABclonal | 1/2000 |  |
| Anti-β-actin antibody | 66009-1-Ig | Proteintech | 1/100000 |  |
| Anti-NDUFS1 antibody | 12444-1-AP | Proteintech | 1/10000 |  |
| Anti-SDHA antibody | A13852 | ABclonal | 1/2000 |  |
| Anti-UQCRC2 antibody | A4366 | ABclonal | 1/2000 |  |
| Anti-COX IV antibody | A6564 | ABclonal | 1/2000 |  |
| Anti-ATP5A1 antibody | A11217 | ABclonal | 1/2000 |  |
| Anti-GLUT4 antibody | A7637 | ABclonal | 1/1000 | 1/100 |
| Anti- Phospho-AKT1-S473 antibody | AP0637 | ABclonal | 1/1000 |  |
| Anti-AKT antibody | 60203-2-Ig | Proteintech | 1/5000 |  |
| Anti- IRS1 antibody | A0245 | ABclonal | 1/1000 |  |
| Anti- Phospho-IRS-1 (Ser307) antibody | #2381 | CST | 1/1000 |  |
| Anti-Flag antibody | #2368 | CST | 1/1000 |  |
| Anti- Phospho-GSK-3β (Ser9) antibody | #5558 | CST | 1/1000 |  |
| Anti-GSK-3β antibody | #9315 | CST | 1/1000 |  |
| Anti-β-Catenin antibody | #8480 | CST | 1/1000 | 1/100 |
| Anti-Phospho-β-Catenin antibody | #9561 | CST | 1/1000 |  |
| Anti-InsR antibody | #3025 | CST | 1/1000 |  |
| AntI-Phospho-InsR(Tyr1150) | #3024 | CST | 1/1000 |  |
| Anti-Wnt3a antibody | #2721 | CST | 1/1000 |  |
| Anti-CILP2 antibody | 11813-1-AP | Proteintech | 1/500 |  |
| Anti-Na^+^-K^+^-ATPase antibody | 14418-1-AP | Proteintech | 1/5000 |  |
| Anti-Lamin B1 antibody | A11495 | ABclonal | 1/1000 |  |
| Anti-CCND1 antibody | 380999 | Zenbio | 1/1000 | 1/100 |
| Anti-Rabbit IgG(H+L) HRP | GAR007 | MultiSciences | 1/100000 |  |
| Anti-Mouse IgG(H+L) HRP | GAM007 | MultiSciences | 1/100000 |  |
| Anti-PCNA antibody | A9909 | ABclonal | 1/1000 | 1/200 |
| Anti-Pax7 antibody | bs-22741R | Bioss |  | 1/100 |
| Anti-Fast Myosin Skeletal Heavy chain antibody | GB112130 | Servicebio |  | 1/1000 |
| Anti-Slow Myosin Skeletal Heavy chain antibody | GB112131 | Servicebio |  | 1/1000 |
| Anti-Myosin heavy chain Type I | BA-D5 | DSHB |  | 1/200 |
| Anti- Myosin heavy chain Type IIA | SC-71 | DSHB |  | 1/200 |
| Anti- Myosin heavy chain Type IIB | BF-F3 | DSHB |  | 1/200 |
| laminin | Ab11575 | Abcam |  | 1/50 |
| Anti-Rabbit IgG(H+L) (Alexa Fluor 594) | ab150084 | Abcam |  | 1/500 |
| Anti-Rabbit IgG(H+L) (Alexa Fluor 488) | ab150077 | Abcam |  | 1/500 |
| CST: Cell Signaling Technology. | | | | |

| **Table S4. Clinical characteristics of patients with or without sarcopenia** | | | |
| --- | --- | --- | --- |
| Variables | Non-sarcopenia (n = 4) | Sarcopenia (n = 4) | p-value |
| Age (years, Mean ± SD) | 75.3±4.23 | 77.8 ±2.58 | 0.451 |
| BMI (kg/m^2^, Mean ± SD) | 22.25±1.24 | 22.75±0.87 | 0.533 |
| Grip strength (kg, Mean ± SD) | 25.88±3.19 | 10.40±1.13 | <0.0001 |
| SMI (kg/m^2^, Mean ± SD) | 6.98±1.12 | 4.38±0.38 | 0.005 |
| Six-meter stride speed(s) | 5.15±0.37 | 11.98±2.22 | <0.001 |
| Five sit-to-stand tests(s) | 9.20±1.18 | 17.08±1.10 | <0.0001 |
| Basal Metabolic Rate (kJ/m²·h) | 1333.54±107.61 | 1105.37±56.73 | 0.009 |
| Laboratory data |  |  |  |
| C-reaction protein(mg/L) | 4.90±1.82 | 8.25±1.58 | 0.032 |
| Leukocyte (10^9 /L) | 5.15±1.08 | 8.80±1.73 | 0.012 |
| SMI: Skeletal Muscle Mass Index; SD: standard deviation. The participants were all female. | | | |

**Supplemental Methods**

**Lentiviral Transfection**

Lentivirus constructs for CILP2-overexpression (OE-CILP2), control-overexpression (OE-GFP), CILP2-shRNA (sh-CILP2), and control shRNA (sh-GFP) were obtained from Genechem Company (Shanghai, China). The sequences for CILP2-targeting shRNA and non-target control shRNA are detailed in **Table S1**. A total of 5×10^4^ cells were plated in 12-well plates; cells at 30-50% confluence were transfected using HitransG Transfection Reagent P (Genechem) according to the manufacturer's instructions. The transfected cells were selected with 4 μg/ml puromycin (ST551-10mg, Beyotime, China) for 10 days to establish stable expressing cells. The effectiveness of transfection was assessed by qRT-PCR and Western blotting.

**Glucose uptake assay**

Glucose uptake assay was performed as described earlier^1^. After the cells were differentiated successfully in the 24-well plate, the medium was discarded and the cells were rinsed three times. Then the medium with the modified medium containing fluorescent glucose analog (Glucose Uptake Probe-Red. Dojindo, Japan) instead of glucose was added. Within 24 h, the glucose uptake capacity of the cells was detected by fluorescence.

**Isolation of plasma membrane fractionation**

The Membrane and Cytosol Protein Extraction Kit (P0033, Beyotime, China) was utilized to isolate plasma membrane proteins from C2C12 myotubes and gastrocnemius tissue following the manufacturer's instructions.

**Nuclear and cytoplasmic protein extraction**

Referring to the previous literature^2^, nuclear and cytoplasmic protein extractions were conducted following the manufacturer's instructions (P0028, Beyotime, China). Briefly, cells grown in 10 cm dishes were washed once with PBS and scraped for centrifugation to obtain a cell pellet. Next, 500 μL of cytoplasmic protein extraction buffer A, pre-mixed with 1 mM PMSF, was added to the cell petri dish and vortexed for several seconds. After 15 minutes, 25 μL of cytoplasmic protein extraction buffer B was added and vortexed for several seconds, followed by centrifugation at 15,000 ×g for 5 minutes. At this stage, the supernatant represents the cytoplasmic fraction. Subsequently, 125 μL of nuclear protein extraction buffer was added to the dish, followed by intermittent vortexing for 30 minutes and centrifugation at 15,000 ×g for 10 minutes. The resulting supernatant is the nuclear fraction.

**Dual-luciferase reporter assay**

C2C12 cells were transfected with a TOPFlash plasmid (D2501, Beyotime, China), which can detect TCF/LEF transcriptional activity with high sensitivity. After 48 hours, firefly and Renilla luciferase activities were measured using a Dual-Luciferase Reporter Assay System (E1910, Promega, USA), according to the manufacturer's protocol. Relative luciferase activity was reported as the ratio of firefly to Renilla luciferase activity.

**Oxygen consumption measurement (Seahorse)**

Oxygen consumption rate assays were conducted as previously described^3^. The Seahorse XF24 extracellular flux analyzer (Seahorse Bioscience, Agilent Technologies, USA) was utilized to evaluate the oxygen consumption rate (OCR) in mitochondria, following the manufacturer-recommended protocols. A total of 30,000 transfected cells were seeded in each well of the microplates. Differentiation commenced the next day with the addition of fusion media for 4 days, after which the cells were cultured for an additional 2 days in post-fusion media. The sensor cartridge was hydrated overnight in XF Calibrant at 37°C in a CO2-free incubator. Subsequently, the cells were washed and incubated with XF-Base Medium (non-buffered DMEM containing 10 mM glucose, 2 mM glutamine, and 1 mM pyruvate, pH 7.4) for 1 hour in an incubator without CO2. OCR values were recorded at baseline and following the addition of 1.5 μM oligomycin, 0.5 µM FCCP, and 0.5 μM rotenone + 0.5 μM antimycin A. OCR values were normalized with cell numbers using Hoechst staining^3^.

**Quantitative Real-Time PCR (qRT-PCR)**

Total RNA from cells or tissues was extracted using TRIzol Reagent (15596026, Invitrogen, USA), followed by cDNA synthesis with a cDNA synthesis kit (RR047Q, Takara, Japan). Real-time PCR was performed with TB Green® Premix Ex Taq™ II (RR820A, Takara, Japan) and StepOnePlus Real-Time PCR System. β-actin served as an internal control. Gene expression was quantified using the 2^−ΔΔCt^ method. The primer sequences are presented in **Table S2**.

**Western Blotting Analysis (WB)**

Skeletal muscle samples or treated C2C12 cells were lysed in ice-cold RIPA buffer containing the PMSF protease inhibitor (ST505, Beyotime, China). Proteins underwent electrophoresis on SDS-PAGE gels with a concentration of 12.5% or 10% (w/v), then transferred onto a PVDF membrane (Bio-Rad, USA). The PVDF membranes were subsequently blocked with 5% nonfat milk before being incubated with primary antibodies at 4°C overnight. Following this, the membranes were washed three times with TBST and incubated with the appropriate secondary antibodies at room temperature for 1 hour. Blots were then visualized using the BeyoECL Plus Chemiluminescence Kit (P0018S, Beyotime, China) and an automatic digital gel/chemiluminescence image analysis system. The primary antibodies utilized for western blot analysis are listed in **Table S3**.

**RNA sequencing and data analysis**

RNA sequencing (RNA‐Seq) and data analysis were performed as previously reported^4^. Total RNA was extracted using TRIzol Reagent (15596026, Invitrogen, USA) according to the manufacturer's protocol. RNA quality was evaluated on an Agilent 2100 Bioanalyzer (Agilent Technologies, Palo Alto, USA) and verified using RNase-free agarose gel electrophoresis. After total RNA extraction, eukaryotic mRNA was enriched with Oligo(dT) beads. The enriched mRNA was then fragmented into short segments using fragmentation buffer and reverse transcribed into cDNA with the NEBNext Ultra RNA Library Prep Kit for Illumina (NEB #7530, New England Biolabs, Ipswich, MA, USA). The purified double-stranded cDNA fragments underwent end repair, an A base was added, and they were ligated to Illumina sequencing adapters. The ligation reaction was purified with AMPure XP Beads (1.0X) and amplified via polymerase chain reaction (PCR). The resulting cDNA library was sequenced using the Illumina Novaseq6000 by Gene Denovo Biotechnology Co. (Guangzhou, China). Finally, the DESeq2 package of R software was utilized for the analysis of differentially expressed genes (DEGs). Genes with P < 0.05 and |Log2FoldChange| ≥1 were classified as DEGs. Additionally, heatmaps and volcano plots of DEGs were generated from the databases using the R programs pheatmap and ggplot2 packages. The Metascape database (http://metascape.org/) was employed for Gene Ontology (GO) analysis.

**Study participants and Biopsies**

The eight patients included in this study were all from the Department of Orthopedics at Fujian Provincial Hospital, awaiting hip replacement due to conditions such as hip fracture, avascular necrosis of the femoral head, and acetabular dysplasia. Exclusion criteria encompassed patients with diseases such as diabetes, hyperthyroidism, secondary osteoporosis, osteomalacia, osteogenesis imperfecta, rheumatoid arthritis, multiple myeloma, bone tumors, or those with a history of using weight-loss drugs or corticosteroids. To eliminate gender differences, all patients were female. For patients with sarcopenia, the diagnosis was primarily based on the Skeletal Muscle Mass Index (SMI) measured by Dual-energy X-ray Absorptiometry (DXA), with the diagnostic criterion defined as low muscle strength (dominant hand grip <18 kg) and low appendicular skeletal muscle mass (SMI ≤ 5.4 kg/m²) according to the 2019 AWGS^5^. Ultimately, four patients with sarcopenia and four without (control group) were included. Samples were collected by a senior chief physician to ensure consistency in sample quality. During the surgery, muscle specimens (2-5g) were taken from the lateral vastus muscle of patients in both groups. The sampling site was located 2 cm anterior and inferior to the lateral lip of the linea aspera of the femur, ensuring the representativeness and comparability of the samples. There were no complications during the biopsy. After removing excess fat and connective tissue, the biopsy specimens were rinsed with cold PBS to flush out the blood. Part of the muscle specimens was fixed in 4% paraformaldehyde for subsequent histological analysis. The remaining muscle specimens were immediately frozen in liquid nitrogen and stored at -80 °C until further analysis. Meanwhile, this study was conducted in accordance with the principles of the Declaration of Helsinki. Approval was granted by the Ethics Committee of Fujian Provincial Hospital (No. K2023-06-007). Informed consent was obtained from individual participants and/or their legal guardians in the study.

**Histological analysis of skeletal muscle**

When the mice were euthanized, the gastrocnemius (GA) muscles were immediately weighed and fixed overnight in 4% paraformaldehyde at 4°C, then embedded in paraffin wax (327204, Sigma-Aldrich, USA). 5 μm paraffin sections were cut, and the slides were stained with hematoxylin and eosin (HE) (C0105M, Beyotime, China), Masson (KGMST-8004, Keygen Biotech, China), and Periodic Acid-Schiff (PAS) (G1008, Servicebio, China) according to the instructions. After the euthanization of the mice, part of the GA muscle was embedded in OCT compound and sectioned at a thickness of 10 μm. The succinate dehydrogenase (SDH) activities were detected using the SDH staining kit (G2000, Solarbio, China). All image analysis was performed using ImageJ processing software.

**Immunofluorescence (IF) Analysis**

Muscle samples were utilized for IF analysis as previously described^24^. The sections were blocked with Quickblock blocking buffer for immune staining (P0260, Beyotime, China) for 15 minutes at room temperature (RT), followed by incubation with primary antibodies at 4 °C overnight. They were then labeled with Alexa Fluor594-preabsorbed goat anti-rabbit IgG (ab150084, Abcam, 1:500, UK) or Alexa Fluor488-preabsorbed goat anti-rabbit IgG (ab150077, Abcam, 1:500, UK) for 2 hours at RT. Subsequently, DAPI staining was conducted for 5 minutes, and the samples were finally visualized using a fluorescence microscope.

**Glycogen content**

Reference to previous literature^6^, we employed the Anthrone-sulfuric acid method to measure the glycogen content in cells or GA muscle. For cell samples, specific reagents were added and centrifuged at high speed, and the supernatant was collected. For fresh muscle samples, after cutting the tissue and adding special reagents, the tissue was boiled at high temperature and then centrifuged at high speed, with the supernatant collected afterward. Glycogen content was quantified using the Glycogen Assay Kit (BC0345, Solarbio, China) following the manufacturer's instructions. Glycogen content (mg/mg protein) was normalized to total protein content (mg/mL) based on the BCA assay (P0010, Beyotime, China).

**Glucose consumption assay**

Cells transfected with various lentiviruses were differentiated into myotubes. The glucose concentration in the medium was measured using the glucose oxidase and peroxidase (GOD-POD) method (A154-1-1, Nanjing Jiancheng Bioengineering Institute, China). The glucose concentration in the wells containing cells was subtracted from that of the blank wells to determine the amount of glucose consumed.

**Cell Counting Kit-8 Assay**

Cell viability was assessed using the Cell Counting Kit-8 (C0038, Dojindo, Japan) following the manufacturer's instructions. Cells transfected with various lentiviruses were seeded into 96-well plates at a density of 4 × 10^3^ cells per well. Ten microliters of CCK-8 solution was added to each well at 0, 24, 48, 72, and 96 hours, and the plates were returned to the incubator for 1 hour. The absorbance of each well was measured at 450 nm using an enzyme-labeled instrument.

**EdU Proliferation Assay**

The 10 μM 5-ethynyl-2′-deoxyuridine (EDU) medium was prepared following the manufacturer's guidelines (C10310-3, C10310-1, RiboBio, China). C2C12 cells transfected with various lentiviruses were seeded into a 24-well plate. Once the C2C12 cells reached the appropriate confluence, the medium was replaced with 100 μL of EDU medium and incubated at 37 ℃ in 5% CO2 for 2 hours. Subsequently, the C2C12 cells were fixed in 4% paraformaldehyde for 20 minutes and incubated with Apollo® reagent (100 μL) for 30 minutes at RT. Afterward, the cells were stained with DAPI dye and examined using a fluorescence microscope. The ratio of EdU-positive cells to the total number of DAPI-positive cells was calculated to assess cell proliferation.

**Co-immunoprecipitation**

Cell lysates (OE-GFP and OE-CILP2) were extracted using a non-denaturing method according to the CO-IP extraction kit's (88804, ThermoFisher, USA) instructions. Briefly, cells grown in 10-cm dishes were washed once with PBS and lysed on ice for 15 minutes with 600 μL lysis buffer. Following centrifugation at 13,000 ×g for 10 minutes at 4°C, the supernatant was divided into 100 μL and 500 μL portions, with the 500 μL incubated with 10 μg antibody overnight at 4°C, while the remaining 100 μL served as the input group. Protein A&G beads were added and incubated for 1 hour at room temperature, followed by washing and elution with SDS-loading buffer at 95°C for 5 minutes. The eluted sample is now ready for Western blot analysis.

Supplemental REFERENCES

1. P. Ji, B. An, Z. Jie, et al., “Genetically Engineered Probiotics as Cata-lytic Glucose Depriver for Tumor Starvation Therapy,” Materials Today Bio 18 (2023): 100515.

2. Y. Cheng, X. Lu, F. Li, et al., “NDFIP1 Limits Cellular TAZ Accumu-lation via Exosomal Sorting to Inhibit NSCLC Proliferation,” Protein & Cell 14 (2023): 123–136.

3. H. Song, X. Tian, D. Liu, et al., “CREG1 Improves the Capacity of the Skeletal Muscle Response to Exercise Endurance via Modulation of Mitophagy,” Autophagy 17 (2021): 4102–4118.

4. W. Qin and J. Weng, “Hepatocyte NLRP3 Interacts With PKCε to Drive Hepatic Insulin Resistance and Steatosis,” Science Bulletin (Beijing) 68 (2023): 1413–1429.

5. L. K. Chen, J. Woo, P. Assantachai, et al., “Asian Working Group for Sarcopenia: 2019 Consensus Update on Sarcopenia Diagnosis and Treatment,” Journal of the American Medical Directors Association 21 (2020): 300–307.e2.

6. W. Ren, J. Chen, W. Wang, et al., “Sympathetic Nerve-Enteroendocrine L Cell Communication Modulates GLP-1 Release, Brain Glucose Utilization, and Cognitive Function,” Neuron 112 (2024): 972–990.e8.

S1. Bahn YJ, Yadav H, Piaggi P, Abel BS, Gavrilova O, Springer DA, et al. CDK4-E2F3 signals enhance oxidative skeletal muscle fiber numbers and function to affect myogenesis and metabolism. *J Clin Invest* 2023;133:

S2. Tumbapo S, Strudwick A, Stastna JJ, Harvey SC, Bloemink MJ. Moderate dietary restriction delays the onset of age-associated sarcopenia in Caenorhabditis elegans due to reduced myosin UNC-54 degradation. *Mech Ageing Dev* 2024;217:111900.

S3. Kirk B, Cawthon PM, Cruz-Jentoft AJ. Global consensus for sarcopenia. *Aging (Albany NY)* 2024;16:

S4. Antuña E, Potes Y, Baena-Huerta FJ, Cachán-Vega C, Menéndez-Coto N, Álvarez Darriba E, et al. NLRP3 Contributes to Sarcopenia Associated to Dependency Recapitulating Inflammatory-Associated Muscle Degeneration. *Int J Mol Sci* 2024;25:

S5. Burton MA, Antoun E, Garratt ES, Westbury L, Dennison EM, Harvey NC, et al. The serum small non-coding RNA (SncRNA) landscape as a molecular biomarker of age associated muscle dysregulation and insulin resistance in older adults. *Faseb j* 2024;38:e23423.

S6. Geng S, Liu SB, He W, Pan X, Sun Y, Xue T, et al. Deletion of TECRL promotes skeletal muscle repair by up-regulating EGR2. *Proc Natl Acad Sci U S A* 2024;121:e2317495121.

S7. Kassar-Duchossoy L, Gayraud-Morel B, Gomès D, Rocancourt D, Buckingham M, Shinin V, et al. Mrf4 determines skeletal muscle identity in Myf5:Myod double-mutant mice. *Nature* 2004;431:466-71.

S8. Qi K, Dou Y, Li C, Liu Y, Song C, Li X, et al. CircGUCY2C regulates cofilin 1 by sponging miR-425-3p to promote the proliferation of porcine skeletal muscle satellite cells. *Arch Anim Breed* 2023;66:285-98.

S9. Sato S, Hanai T, Kanamoto T, Kawano F, Hikida M, Yokoi H, et al. Vibration acceleration enhances proliferation, migration, and maturation of C2C12 cells and promotes regeneration of muscle injury in male rats. *Physiol Rep* 2024;12:e15905.

S10. Kim JW, Kim J, Cho JY, Shin Y, Son H, Sathiyamoorthy S, et al. Association Between Muscle Growth and Transcription of a Mutant MSTN Gene in Olive Flounder (Paralichthys olivaceus). *Mar Biotechnol (NY)* 2024;

S11. Rom O, Reznick AZ. The role of E3 ubiquitin-ligases MuRF-1 and MAFbx in loss of skeletal muscle mass. *Free Radic Biol Med* 2016;98:218-30.

S12. Ye YL, Kuai Z, Qian DD, He YT, Shen JP, Wu KF, et al. GLP-2 ameliorates D-galactose induced muscle aging by IGF-1/Pi3k/Akt/FoxO3a signaling pathway in C2C12 cells and mice. *Arch Gerontol Geriatr* 2024;124:105462.

S13. Burton MA, Garratt ES, Hewitt MO, Sharkh HY, Antoun E, Westbury LD, et al. DNA methylation of insulin signaling pathways is associated with HOMA2-IR in primary myoblasts from older adults. *Skelet Muscle* 2023;13:17.

S14. Shalit A, Gerontiti E, Boutzios G, Korakianiti E, Kanouta F, Vasileiou V, et al. Nutrition of aging people with diabetes mellitus: Focus on sarcopenia. *Maturitas* 2024;185:107975.

S15. Prabakaran AD, McFarland K, Miz K, Durumutla HB, Piczer K, El Abdellaoui-Soussi F, et al. Intermittent glucocorticoid treatment improves muscle metabolism via the PGC1α/Lipin1 axis in an aging-related sarcopenia model. *J Clin Invest* 2024;

S16. Yang S, Yang G, Wang X, Li L, Li Y, Xiang J, et al. MicroRNA-92b in the skeletal muscle regulates exercise capacity via modulation of glucose metabolism. *J Cachexia Sarcopenia Muscle* 2023;14:2925-38.

S17. Guo M, Zhang J, Ma Y, Zhu Z, Zuo H, Yao J, et al. AAV-Mediated nuclear localized PGC1α4 delivery in muscle ameliorates sarcopenia and aging-associated metabolic dysfunctions. *Aging Cell* 2023;22:e13961.

S18. Tezze C, Sandri M, Tessari P. Anabolic Resistance in the Pathogenesis of Sarcopenia in the Elderly: Role of Nutrition and Exercise in Young and Old People. *Nutrients* 2023;15:

S19. Dreher SI, Grubba P, von Toerne C, Moruzzi A, Maurer J, Goj T, et al. IGF1 promotes human myotube differentiation toward a mature metabolic and contractile phenotype. *Am J Physiol Cell Physiol* 2024;326:C1462-c81.

S20. Li Q, Pu D, Xia X, Liu H, Li L. Serum Concentrations of Cartilage Intermediate Layer Protein 2 Were Higher in Overweight and Obese Subjects. *Biomed Res Int* 2022;2022:6290064.

S21. Ahmad SS, Chun HJ, Ahmad K, Choi I. Therapeutic applications of ginseng for skeletal muscle-related disorder management. *J Ginseng Res* 2024;48:12-9.

S22. Prajapati P, Kumar A, Mangrulkar S, Chaple DR, Saraf SA, Kushwaha S. Azilsartan prevents muscle loss and fast- to slow-twitch muscle fiber shift in natural ageing sarcopenic rats. *Can J Physiol Pharmacol* 2024;102:342-60.

S23. Cho HJ, Kim H, Lee YS, Moon SA, Kim JM, Kim H, et al. SLIT3 promotes myogenic differentiation as a novel therapeutic factor against muscle loss. *J Cachexia Sarcopenia Muscle* 2021;12:1724-40.

S24. Wajda A, Bogucka D, Stypińska B, Radkowski MJ, Targowski T, Dudek E, et al. Expression of Prostaglandin Genes and β-Catenin in Whole Blood as Potential Markers of Muscle Degeneration. *Int J Mol Sci* 2023;24:

S25. Brack AS, Conboy MJ, Roy S, Lee M, Kuo CJ, Keller C, et al. Increased Wnt signaling during aging alters muscle stem cell fate and increases fibrosis. *Science* 2007;317:807-10.

S26. DeCarolis NA, Wharton KA, Jr., Eisch AJ. Which way does the Wnt blow? Exploring the duality of canonical Wnt signaling on cellular aging. *Bioessays* 2008;30:102-6.

S27. Wang Y, Mohamed JS, Alway SE. M-cadherin-inhibited phosphorylation of ß-catenin augments differentiation of mouse myoblasts. *Cell Tissue Res* 2013;351:183-200.

S28. Gessler L, Huraskin D, Eiber N, Hashemolhosseini S. The impact of canonical Wnt transcriptional repressors TLE3 and TLE4 on postsynaptic transcription at the neuromuscular junction. *Front Mol Neurosci* 2024;17:1360368.

S29. Fujimaki S, Wakabayashi T, Takemasa T, Asashima M, Kuwabara T. The regulation of stem cell aging by Wnt signaling. *Histol Histopathol* 2015;30:1411-30.

S30. Casas-Fraile L, Cornelis FM, Costamagna D, Rico A, Duelen R, Sampaolesi MM, et al. Frizzled related protein deficiency impairs muscle strength, gait and calpain 3 levels. *Orphanet J Rare Dis* 2020;15:119.

S31. Shin DI, Jin YJ, Noh S, Yun HW, Park DY, Min BH. Exosomes Secreted During Myogenic Differentiation of Human Fetal Cartilage-Derived Progenitor Cells Promote Skeletal Muscle Regeneration through miR-145-5p. *Tissue Eng Regen Med* 2024;21:487-97.

S32. Wang J, Cui C, Chim YN, Yao H, Shi L, Xu J, et al. Vibration and β-hydroxy-β-methylbutyrate treatment suppresses intramuscular fat infiltration and adipogenic differentiation in sarcopenic mice. *J Cachexia Sarcopenia Muscle* 2020;11:564-77.

S33. Richardson K. Genes and knowledge: Response to Baverstock, K. the gene an appraisal. <https://doi.org/10.1016/j.pbiomolbio.2021.04.005>. *Prog Biophys Mol Biol* 2021;167:12-7.

S34. Šedová L, Pravenec M, Křenová D, Kazdová L, Zídek V, Krupková M, et al. Isolation of a Genomic Region Affecting Most Components of Metabolic Syndrome in a Chromosome-16 Congenic Rat Model. *PLoS One* 2016;11:e0152708.

S35. Liu Q, Liao L. Identification of macrophage-related molecular subgroups and risk signature in colorectal cancer based on a bioinformatics analysis. *Autoimmunity* 2024;57:2321908.

S36. Liu D, He C, Liu Z, Xu L, Li J, Zhao Z, et al. The Prognostic and Immune Significance of CILP2 in Pan-Cancer and Its Relationship with the Progression of Pancreatic Cancer. *Cancers (Basel)* 2023;15:

S37. Wang J, Du J, Wang Y, Song Y, Wu J, Wang T, et al. CILP2 promotes hypertrophic scar through Snail acetylation by interaction with ACLY. *Biochim Biophys Acta Mol Basis Dis* 2024;1870:167202.

S38. Piao L, Huang Z, Inoue A, Kuzuya M, Cheng XW. Human umbilical cord-derived mesenchymal stromal cells ameliorate aging-associated skeletal muscle atrophy and dysfunction by modulating apoptosis and mitochondrial damage in SAMP10 mice. *Stem Cell Res Ther* 2022;13:226.

S39. von Haehling S, Coats AJS, Anker SD. Ethical guidelines for publishing in the Journal of Cachexia, Sarcopenia and Muscle: update 2021. *J Cachexia Sarcopenia Muscle* 2021;12:2259-61.
